# Exploring the Anticancer Activity of Tamoxifen-Based Metal Complexes Targeting Mitochondria

**DOI:** 10.1021/acs.jmedchem.3c00617

**Published:** 2023-07-06

**Authors:** Valeria Scalcon, Riccardo Bonsignore, Jana Aupič, Sophie R. Thomas, Alessandra Folda, Alexandra A. Heidecker, Alexander Pöthig, Alessandra Magistrato, Angela Casini, Maria Pia Rigobello

**Affiliations:** † Department of Biomedical Sciences, University of Padova, Via Ugo Bassi 58/b, 35131 Padova, Italy; ‡ Dipartimento di Scienze e Tecnologie Biologiche, Chimiche e Farmaceutiche, Università degli Studi di Palermo, Viale delle Scienze, Edificio 17, 90128 Palermo, Italy; § National Research Council of Italy Institute of Materials (CNR-IOM) C/o SISSA, Via Bonomea 265, 34136 Trieste, Italy; ∥ Chair of Medicinal and Bioinorganic Chemistry, Department of Chemistry, School of Natural Sciences, Technical University of Munich, Lichtenbergstraße 4, D-85748 Garching bei, München, Germany; ⊥ Catalysis Research Center & Department of Chemistry, Chair of Inorganic and Metal-Organic Chemistry, School of Natural Sciences, Technical University of Munich, Ernst-Otto-Fischer Str. 1, D-85748 Garching bei, München, Germany

## Abstract

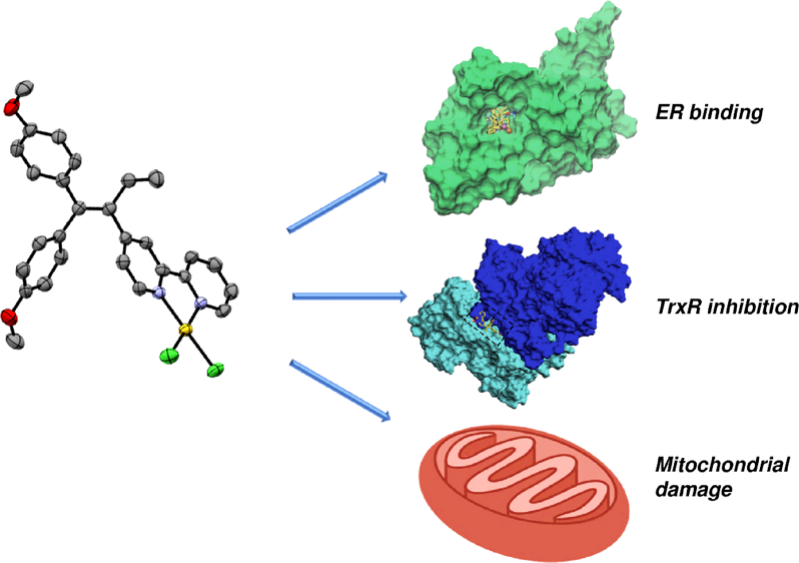

Two new ‘hybrid’ metallodrugs of Au(III) (AuTAML) and Cu(II) (CuTAML) were designed featuring a tamoxifen-derived pharmacophore to ideally synergize the anticancer activity of both the metal center and the organic ligand. The compounds have antiproliferative effects against human MCF-7 and MDA-MB 231 breast cancer cells. Molecular dynamics studies suggest that the compounds retain the binding activity to estrogen receptor (ERα)*. In vitro* and *in silico* studies showed that the Au(III) derivative is an inhibitor of the seleno-enzyme thioredoxin reductase, while the Cu(II) complex may act as an oxidant of different intracellular thiols. In breast cancer cells treated with the compounds, a redox imbalance characterized by a decrease in total thiols and increased reactive oxygen species production was detected. Despite their different reactivities and cytotoxic potencies, a great capacity of the metal complexes to induce mitochondrial damage was observed as shown by their effects on mitochondrial respiration, membrane potential, and morphology.

## Introduction

**Scheme 1:**
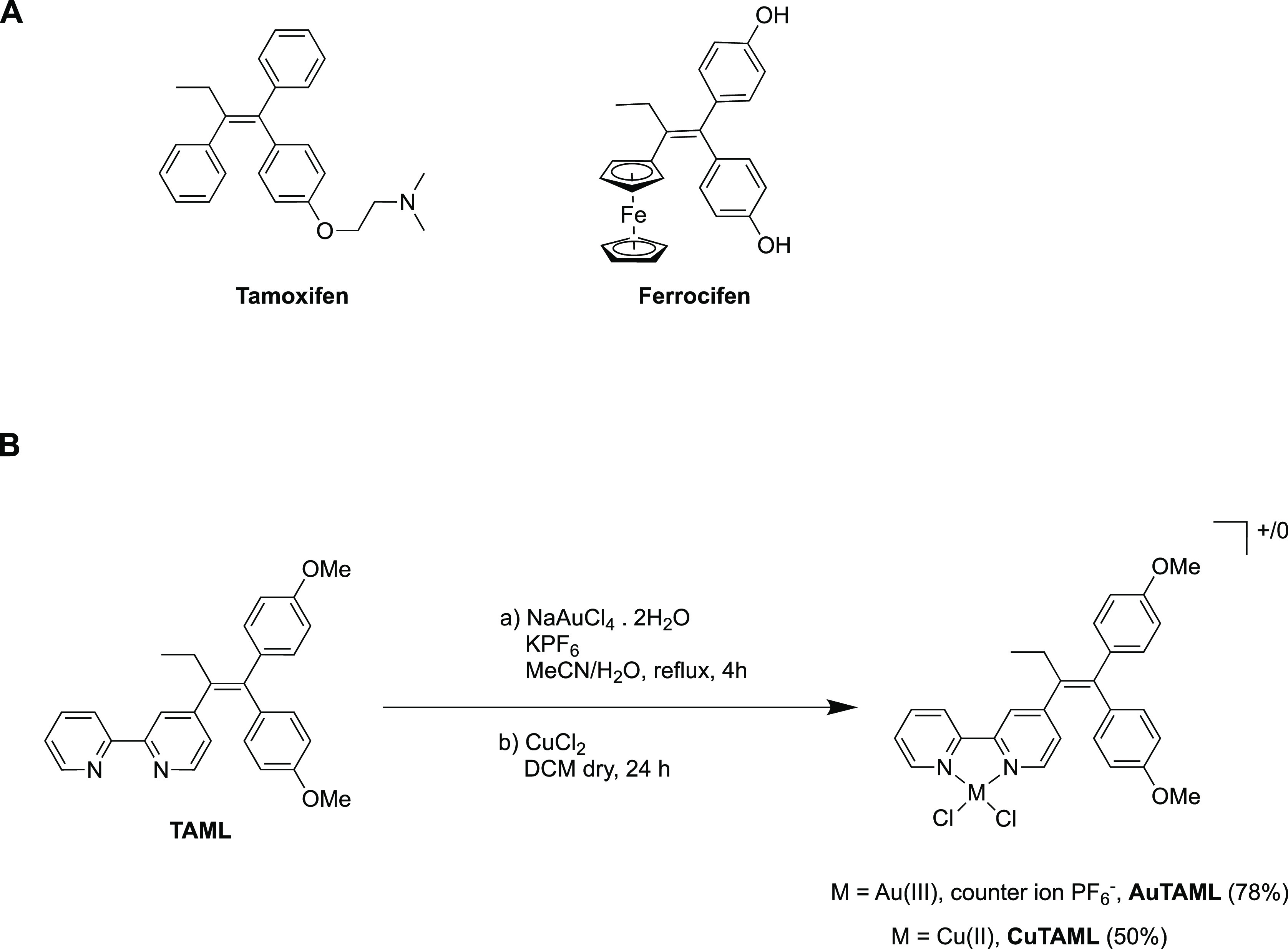
(A) Structures of Tamoxifen and Ferrocifen; (B) Synthetic Pathways to the Au(III) Complex AuTAML and the Cu(II) Complex CuTAML

Breast cancer is a predominant form of cancer among western women, with an incidence of one case per eight women.^1^ More than 60% of breast cancers are estrogen receptor (ER)-positive.^2^ The standard reference for endocrine therapy related to this disease is based on targeting aromatase, which performs estrogen biosynthesis, or via selective estrogen receptor modulators (SERM) or degraders (SERD). Among the SERMS, tamoxifen (Scheme 1) was introduced in cancer treatment during the early 70s.^3,4^ The antitumor effects of tamoxifen are primarily attributable to the modulation of gene expression by competing with estrogen binding and by acting as an antagonist of the estrogen receptor, thereby leading to inhibition of proliferation and increased apoptosis of breast cancer cells.^5,6^ However, the efficacy of this drug is not satisfactory due to the development of resistance after prolonged therapeutic regimens and disease relapse.^7,8^ Furthermore, tamoxifen treatment stimulates proliferation of different tissues, and known tamoxifen side effects comprise endometrial hyperplasia, venous thromboembolic disease, and even hepatic toxicity.^9−12^

In the search for more potent and mechanism-oriented anticancer drugs, a ‘hybrid’ medicinal chemistry approach exploiting the unique behavior of transition-metal complexes jointed with clinically applied pharmacophores may provide a real advancement. In fact, in recent years, several successful examples of metal–organic scaffolds, featuring established drug molecules, have been reported for their therapeutic potential in cancer treatment.^13−20^ One prominent example of this hybrid approach related to tamoxifen is the ferrocene-containing derivative ferrocifen (Fc-diOH) (Scheme 1), which was designed and extensively biologically tested by Jaouen and coworkers together with several analogues.^21,22^ The mechanistic studies show that ferrocifens are activated in cancer cells by oxidation with generation of organometallic quinone methide species,^23^ shown to react with thiols via a 1,8-Michael addition.^24,25^ The resulting quinone methides were reported to inhibit the selenoenzyme thioredoxin reductase (TrxR),^25−27^ responsible within the thioredoxin system for maintaining the intracellular redox balance^28^ which is an established anticancer drug target.^29−31^ Of note, ferrocifens showed redox chemistry^32^ and high activity toward both hormone-dependent (e.g., MCF-7) and hormone-independent (e.g., MDA-MB 231) human cancer cell lines.^22^ Concerning other tamoxifen-based metallodrugs, platinum, titanium, and ruthenium derivatives were reported for their anticancer effects.^33−36^ In 2019, Hey-Hawkins and coworkers described the antiproliferative effects of compounds featuring half-sandwich molybdacarboranes.^37^ These complexes were found to have diverse biological effects compared to Fc-diOH, including induction of senescence and autophagy.^37^ Recently, the same group reported on Pt(II) and Pd(II) derivatives with the same tamoxifen-related ligand.^38^

Inspired by these promising results and to enhance the multitargeting activities of tamoxifen-based metallodrugs, we have synthesized two coordination complexes of Au(III) (AuTAML) and Cu(II) (CuTAML) with the ligand 4-[1,1-bis(4-methoxyphenyl)but-1-en-2-yl]-2,2′-bipyridine (TAML)^37^ (Scheme 1), a derivative of tamoxifen incorporating a 2,2′-bipyridine moiety as a metal binding bidentate N^N domain. The choice of including gold into the compound’s scaffold stems from the already well-established anticancer activity of Au(III) complexes *in vitro* and *in vivo*,^39−41^ often related to the metallodrug activation either by reduction or ligand substitution mechanisms,^41,42^ and attributable to the targeting of several metabolic pathways and a number of metabolites in tumor cells.^43^ Numerous studies report on the affinity of gold complexes for thioredoxin reductases (TrxRs).^31,43−45^ Mammals present three types of TrxRs: the cytosolic (TrxR1) and the mitochondrial (TrxR2) isoforms, both highly expressed in cancer cells,^46,47^ and a third isoenzyme highly expressed in testis, thioredoxin-glutathione reductase (TrxR3).^48^ Human TrxRs are homodimers coupled head to tail, each one containing a redox-active selenocysteine residue (Sec-498) in a ligand-accessible active site that allows for the interaction with various substrates and with thioredoxin.^31,49^ Au(I)/(III) ions (soft acids) can easily access this region, resulting in strong binding to selenolate (soft base) while a ligand exchange reaction takes place, displacing a ligand from the gold compound.^42,50^ The so-formed gold-selenide adduct cannot function as a disulfide redox-active center within TrxR, and consequently, the enzyme’s activity is inhibited. Inhibition of TrxR causes important mitochondrial damage, eventually inducing apoptosis. In fact, it drives an increase in the intracellular concentration of reactive oxygen species (ROS), leading to the opening of the mitochondrial permeability transition pore as well as altering the permeability of the mitochondrial membrane and stimulating cytochrome c release.^31,51,52^ Interestingly, tamoxifen exhibits ER-independent anticancer effects in various cancer cell types for which induction of apoptosis through mitochondrial dysfunction has been advocated as one of the mechanisms underlying such effects.^53^

Concerning the copper-tamoxifen derivative (CuTAML), it should be noted that an important number of copper compounds have been studied as experimental anticancer agents *in vitro* and, in some cases, *in vivo* showing pharmacological effects against different cancer types.^54−57^ While the mechanisms of cytotoxic action of these metallodrugs have not been fully elucidated and may also involve effects attributable to the copper coordinating ligands, the obtained results point toward a multimodal spectrum of biological activities, involving intracellular redox reactions triggered by the metal centre.^58,59^ In general, copper homeostasis can be leveraged as a cancer vulnerability, where the two major current treatment approaches targeting this nutrient include either chelators to deplete copper pools that drive tumor proliferation and metastasis, or copper ionophores to supplement copper ions and drive cuproptosis, an oxidative stress-inducing form of cell death triggered by copper excess.^60^

Overall, our design strategy is centered on such tamoxifen-metal-hybrid prodrugs, which would (i) release both pharmacophores and their active species inside cancer cells and (ii) ensure their synergistic anticancer action. The compounds have been characterized by various methods, and their stability in aqueous solution has been assessed by UV–visible spectrophotometry. They were further tested for their antiproliferative activity in two human breast cancer cell lines, namely, MDA-MB 231 and MCF-7 cells, which differ in the expression of estrogen (ER)/progesterone (PR) receptors and human epidermal growth factor receptor type 2. While MDA-MB 231 cells are considered triple negative since they lack all three receptor types, MCF-7 cells are hormone-dependent (ER^+^/PR^+^). Mechanistic insights highlighting differences among the metal complexes and their free ligand have been obtained via different biochemical assays. Specifically, the research activity was conducted both on isolated enzymes, *in silico* and in cancer cell models. First, in order to explore the effects of the ligand itself and of the metal complexes, we performed enzymatic activity estimations and interaction assays on the isolated proteins thioredoxin reductase and glutathione reductase (GR), which is a homolog antioxidant enzyme without Sec. In parallel, molecular dynamics (MD) simulations were applied to investigate the binding modes of the new metal complexes and free ligand to the estrogen receptor alpha (ERα) in comparison to tamoxifen. Further, MD studies were conducted to characterize the covalent adduct of the Au(III) complex with the Sec catalytic residue of human TrxR1. Afterward, the potential enzyme inhibitory activity of the new complexes was tested in cancer cells as well as their effect on redox pathways and on mitochondria via several complementary approaches.

## Results and Discussion

### Synthesis and Characterization

The ligand TAML was prepared in a 4-steps synthesis (Scheme S1) as described by Hey-Hawkins and coworkers,^37^ with the eluent mixtures for the column chromatography adapted accordingly, to afford highly pure compounds. Thus, the 2,2′-bipyridine was achieved by Stille coupling upon alkylation of the isonicotinic acid with ethylmagnesium bromide. Subsequently, the dichloro-olefination of the carbonyl moiety followed by the [Pd(PPh_3_)_4_]-catalyzed Suzuki coupling afforded the ligand TAML.

To synthesize the Au(III) complex AuTAML, one equivalent of the ligand was reacted for 4 h in MeCN/H_2_O with sodium tetrachloroaurate dihydrate in the presence of an excess of KPF_6_ (Scheme 1). The filtration of the cooled mixture yielded AuTAML as an orange solid (78% yield). NMR analyses of the product (Figures S1–S5) confirmed the complexation of the ligand, as shifted signals compared to TAML appeared, which is particularly evident for the two protons in ortho to the nitrogen atoms (H1 and H7 in Figure S1) shifting from 8.7 and 8.5 to 9.4 and 9.0 ppm, respectively. HR-ESI-MS and elemental analysis further confirmed the achievement and purity of the Au(III) complex (Figure S6).

TAML complexation with Cu(II) was afforded by directly reacting the ligand with 1 equivalent of dry CuCl_2_ under inert conditions for 24 h (Scheme 1). Upon solvent evaporation, further redissolution in MeCN followed by the addition of an excess of diethylether allowed the obtainment of CuTAML in good yield (50%) as a green precipitate. Elemental analysis on the powder confirmed the purity of the compound with the HR-ESI-MS showing the presence of the species [ML-Cl]^+^ at 520.0957 *m*/*z* (Figure S7).

**Figure 1:**
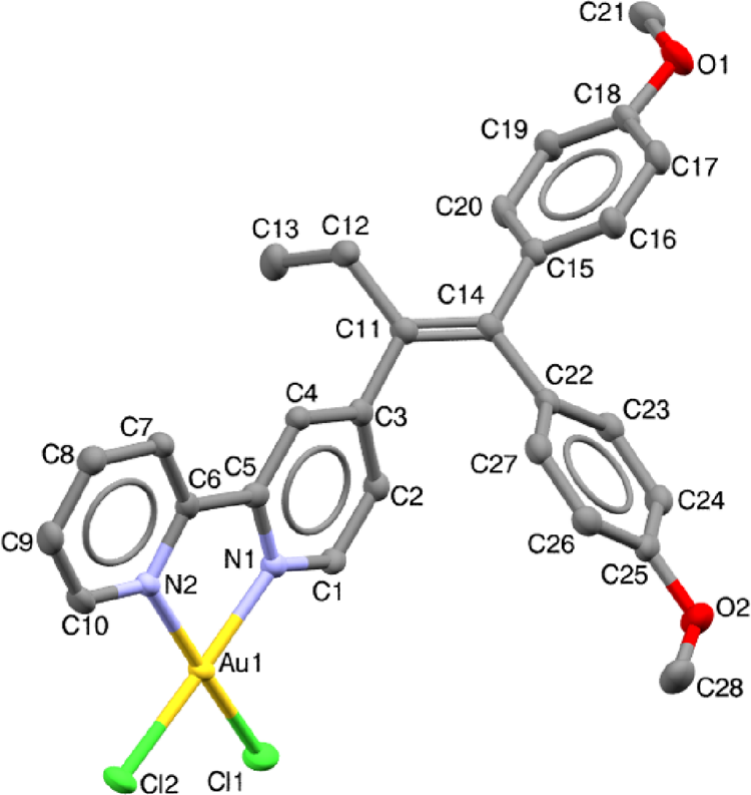
X-ray diffraction analysis. Molecular structure of AuTAML with ellipsoids set at the 50% probability level. Hydrogens have been omitted for clarity.

Crystals of AuTAML were grown by slow evaporation of a saturated solution in MeCN. The structure was determined by X-ray diffraction (XRD) analysis (see Table S1). The solid-state molecular structure of the cation is depicted in Figure 1. The compound crystallizes in the *C*1*c*1 monoclinic space group, and the asymmetric unit contains the AuTAML cation and a AuCl_2_^–^ anion (Figure S8). The coordination of the gold atom with the TAML is almost regular square-planar, with a slight distortion in the N–Au–N angle: 80.98°. The bond lengths and angles involving the gold atom are comparable to those observed in a simple 4,4′-dimethyl-2,2′-bipyridine Au(III) complex.^61^ The Au(III) bipyridine moiety of AuTAML remains coplanar; however, the two phenyl rings of the TAML point out of plane, which was also observed in the ligand and half-sandwich molybdacarborane complex of Hey-Hawkins and coworkers.^37^

To assess the compounds’ stability in an aqueous environment, the absorbance of the compounds’ solutions in 1× PBS buffer (pH 7.4) was measured at room temperature between 250 and 800 nm at regular time intervals for 24 h, allowing the monitoring of possible compound transformations such as hydrolysis, reduction, and/or precipitation. Both the metal complexes’ spectra (Figures S9 and S10) show absorption bands at ca. 300 nm, likely related to a π–π* transition of TAML (see Figure S11), while ligand-to-metal charge transfer transitions appear at 413 and 357 nm for AuTAML and CuTAML, respectively. The Au(III) compound shows a remarkable stability over 24 h with less than a 3% spectral variation over time that could be attributed to partial hydrolysis of the chloride ligands. In fact, several studies demonstrated that Au(III) complexes can easily hydrolyze the chlorido ligands in physiological conditions. Moreover, experimental studies and calculations highlighted mixed chloro-hydroxo/aquo species to be the dominant ones for the reaction with endogenous targets.^62,63^ Concerning CuTAML, a much more complicated pattern appears when studying the stability of the compound in PBS buffer at room temperature, whereby the intensity of the band at 357 nm experiences a 66% hypochromic effect over 24 h, which is likely due to the precipitation of the compound in solution over time (see Figure S9).

**Figure 2:**
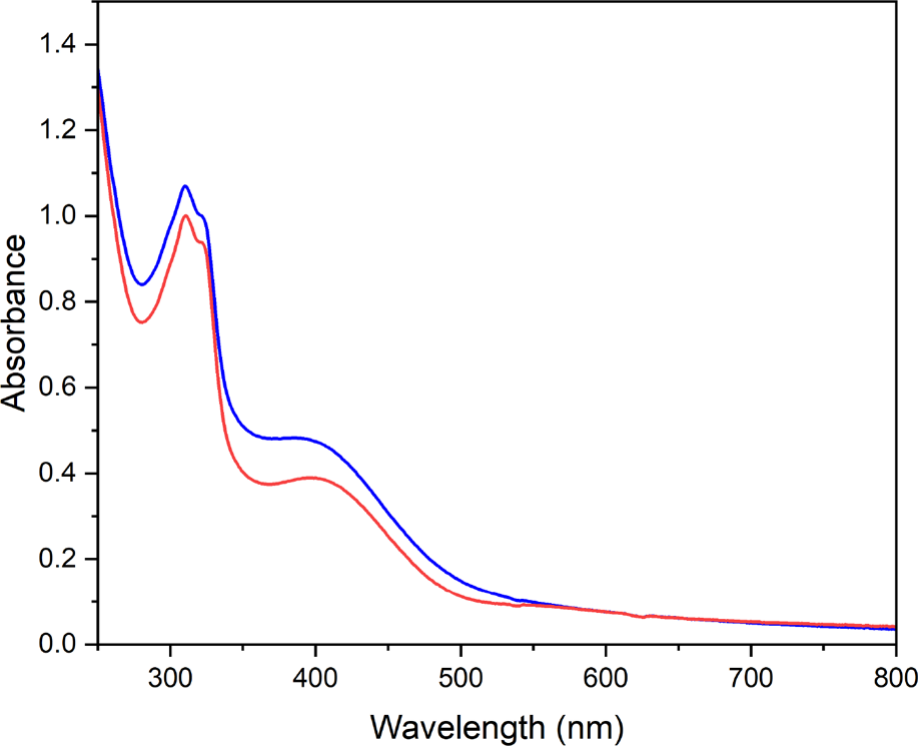
Stability studies by UV–vis absorption spectroscopy. UV–vis spectra of AuTAML (60 μM) in 1× PBS (pH 7.4) at 37 °C. Spectra recorded at *t* = 0 (blue trace) and 24 h (red trace).

The stability of AuTAML was also recorded in the same buffer at 37 °C (Figure 2), showing only a moderate hypochromic shift over 24 h and pointing toward high stability in physiological conditions. Of note, addition of excess glutathione (GSH) to a solution of AuTAML, following previously reported procedures,^64^ also showed high stability of the compound in PBS (pH 7.4) at 37 °C (see Figure S12). In general, the observed spectral changes may be related to the occurrence of partial hydrolysis processes, but the classical LMCT (ligand-to-metal charge-transfer) bands characteristic of the Au(III) chromophore remained well defined over 24 h. Formation of colloidal gold could also be excluded due to the lack of the characteristic absorption bands around 550 nm.

### Antiproliferative Effects

The metal compounds’ antiproliferative activity was tested in the two breast cancer cell lines (MDA-MB 231 and MCF-7) after 48 h in comparison to the free ligand TAML by an MTT assay (see the Experimental section for details). Prior to this analysis, the stability of the compounds in CD_3_CN/D_2_O (80/20) was assessed over 24 h by ^1^H NMR spectroscopy (see representative spectra in Figure S13). Accordingly, MeCN was used to prepare stock solutions of the compounds as described in the Experimental section.

The obtained results show that all the compounds are more effective against the ER^+^ (MCF-7) cells vs the ER^–^ (MDA-MB 231) ones (Table 1) as expected. While AuTAML and TAML have similar EC_50_ values as the benchmark tamoxifen in MCF-7 cells (ca. 8 μM), CuTAML displays higher anticancer potency (EC_50_ = 3 μM). Of note, all the new compounds are more active than cisplatin in the tested conditions. Concerning AuTAML, its intracellular reduction by biological nucleophiles, leading to TAML ligand release, cannot be excluded, in line with the observed similar antiproliferative activities. It is worth mentioning that recently reported Pt(II)- and Pd(II)-TAML analogues showed antitumor activity in the same cancer cell lines, which were attributed to off-target mechanisms rather than only ERα inhibition, for which these compounds were originally designed.^38^

**Table 1:** Cytotoxicity of the TAML Complexes on Breast cancer Cells^c^

EC_50_ (μM)	AuTAML	CuTAML	TAML	tamoxifen	cisplatin
MCF-7 (ER^+^)	7.5 ± 2.4**	3.1 ± 0.9**	8.0 ± 2.2**	8.0 ± 0.7^a^	20.7 ± 1.1^b^
MDA-MB 231 (ER^–^)	23.5 ± 3.1*	6.0 ± 1.1**	21.0 ± 3.5*	15.2 ± 2.3^a^	63.1 ± 1.2^b^

a From ref (65).

b From ref (66).

c EC_50_ values of cytotoxicity of the new compounds after 48 h incubation with respect to the parent molecule tamoxifen and cisplatin in ER^+^ (MCF-7) and ER^–^ (MDA-MB 231) human breast cancer cells. Mean ± SD of 3 experiments is reported (*p < 0.05; **p < 0.01 compared to negative control).

### Molecular Dynamics Studies of the Metal Complexes’ Adducts with ERα

**Figure 3:**
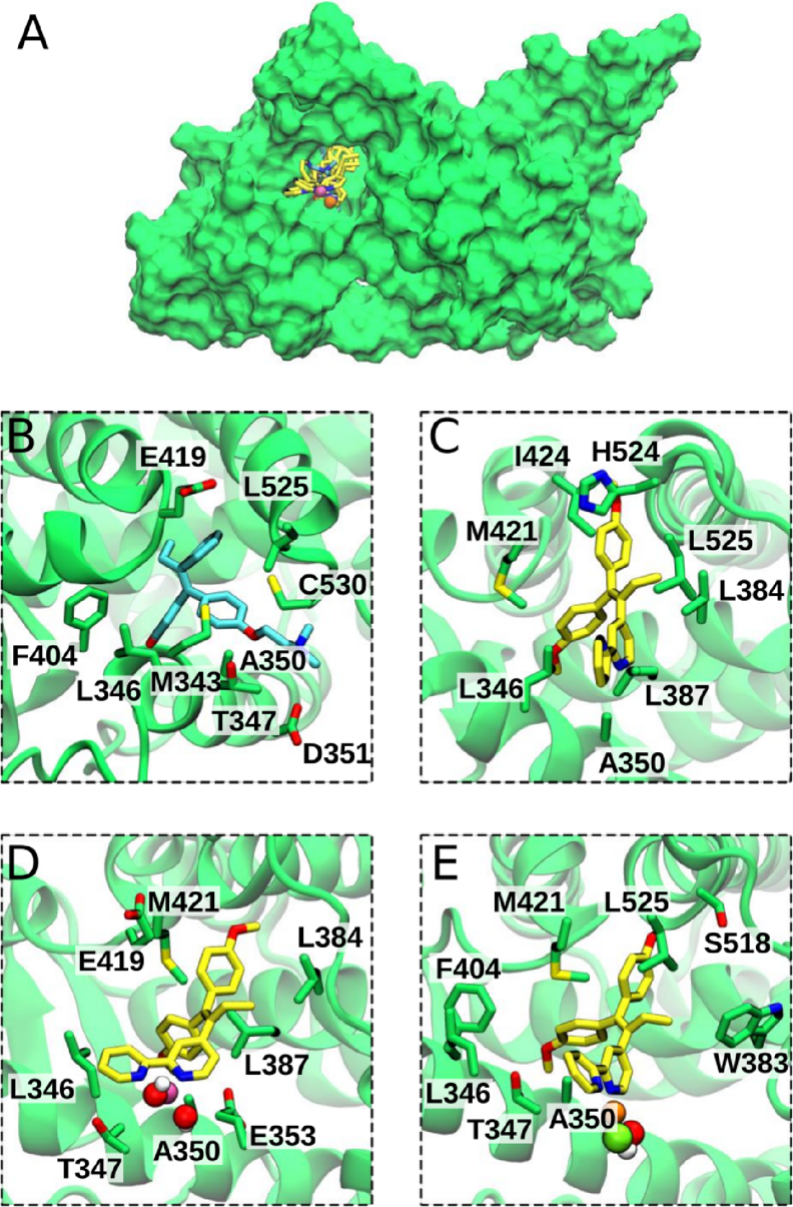
Compounds docked into the ERα ligand binding domain (LBD) and refined by molecular dynamics (MD) simulations. ERα LBD is shown in green surface (A) and cartoon style in adducts with 4-hydroxytamoxifen (OHT), TAML, [CuTAML(OH)_2_], and [AuTAML(*OH*)Cl] (B–E). (A) The overlay of the metal complexes and TAML shows that they occupy the same binding pocket as OHT. (B−E) Close-up of the ERα binding pocket after MD simulations with OHT (B), TAML (C), [CuTAML(OH)_2_] (D), and [AuTAML(*OH*)Cl] (E). Carbon atoms are shown either as cyan (OHT) or yellow licorice (all the other compounds), while the other atoms are shown in red (oxygen), blue (nitrogen), and white (hydrogen). Copper, gold, and chloride are depicted as pink, orange, and light green van der Waals spheres. Residues with the highest contribution to the binding energies are shown in green licorice. As the starting point of ligand docking simulations, we used the structure of the ligand binding domain of ERα when in complex with OHT (PDB ID: 3ERT).

Since the target of tamoxifen is ERα, we examined the binding mode of the new tamoxifen derivatives to this receptor and compared it to that of the active metabolite of tamoxifen, i.e., 4-hydroxytamoxifen (OHT). The binding of OHT to ERα is well-established, since the structure of the complex between OHT and the ERα ligand binding domain (LBD) was resolved by X-ray crystallography.^67^ We performed docking simulations of the metal complexes in the estrogen binding cavity of ERα LBD. Considering the stability of the metal complexes previously detailed, we used the most likely species forming in aqueous solution: in the case of AuTAML, we considered the complex after hydrolysis of one chlorido ligand, while for CuTAML, we modeled the hydrolysis of both chlorido ligands. Each metal complex/ERα LBD system was relaxed by performing 300 ns long classical MD simulation. As a result, we observed that the TAML portion of both metal complexes snugly fits within the ligand binding cavity of ERα (Figure 3A). Thus, these compounds, binding to the antagonist conformation of the ERα, most likely block the estrogen-induced cellular proliferation.^68^ Although their orientation within the cavity is slightly different (Figure 3B–E), all compounds are stabilized by establishing hydrophobic interactions with the M421, L525, and L346 residues, as observed for OHT. Consistently, owing to the hydrophobic nature of the ligand binding cavity, the hydrophilic moiety of the metal-coordinating compound is exposed toward the solvent and does not establish any interaction with ERα. Calculation of the binding free energy (Δ*G*_b_, Table 2) revealed that, for the newly designed compounds, the values in the order of −20 kcal/mol are similar to OHT. This further supports the idea that the antiproliferative activities observed experimentally are at least partly related to the ability of these compounds to target ERα.

**Table 2:** Estimation of the Binding Free Energies on ERα^a^

	OHT	TAML	[CuTAML(OH)_2_]	[AuTAML(*OH*)Cl]
Δ*G*_b_	–21.3 ± 4.3	–24.9 ± 3.3	–21.7 ± 3.8	–23.3 ± 5.0

a Binding free energies (Δ*G*_b_, kcal/mol) of 4-hydroxytamoxifen (OHT), TAML, [CuTAML(OH)_2_], and [AuTAML(*OH*)Cl] to ERα as calculated with the MM-PBSA method. Standard deviation is reported.

### Interaction between Metal TAML Complexes and Isolated Redox Enzymes

**Figure 4:**
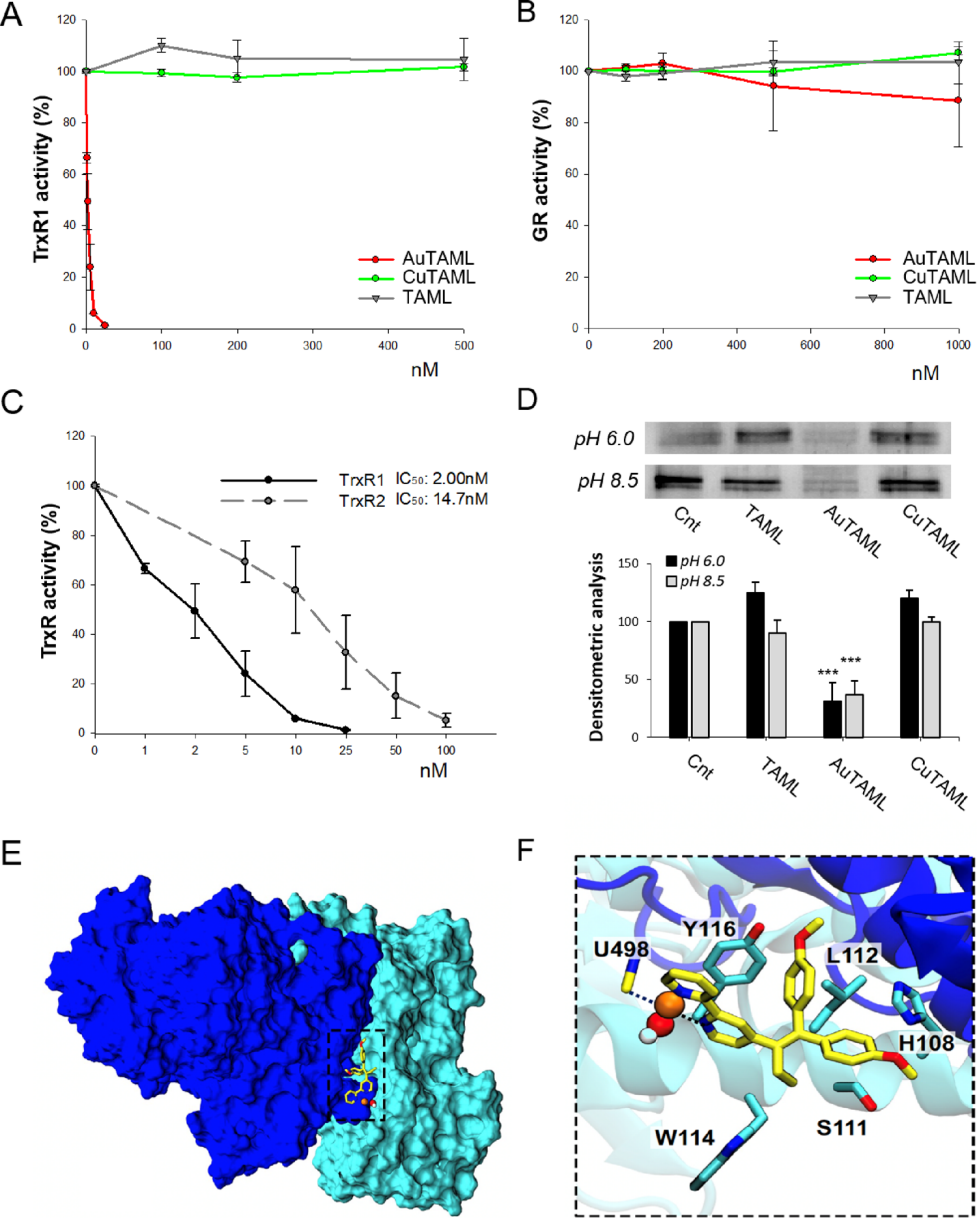
Effect of the complexes on the isolated enzymes *in vitro*. Purified TrxR1 (A) and GR (B) were incubated with increasing concentrations of the metal complexes or TAML alone and the enzymatic activity was evaluated spectrophotometrically, as described in the Experimental section. (C) Comparison of the inhibitory effect of AuTAML on the enzymatic activities of TrxR1 (cytosolic isoform) and TrxR2 (mitochondrial isoform). Mean values ± SD of 3 experiments are reported. (D) Biotinylated iodoacetamide (BIAM) assay on TrxR1 treated with the metal complexes and free ligand (10 μM) for 30 min at room temperature. The band intensity is inversely correlated with the ability of the compounds to bind to the selenocysteine (pH 6.0) or cysteines (pH 8.5) present in TrxR1. Densitometric analysis of the bands was performed using ImageJ software. Mean values ± SD of 2 experiments are reported. ***p < 0.001. (E, F) AuTAML covalently bound to human TrxR1 Sec-498 (U498). The homodimeric TrxR1 protein is depicted as light- and dark-blue for each monomer (TrxR, PDB ID:2J3N) (E). During MD simulations, the compound covalently bound to the C-terminal of the protein transiently intercalated at the homodimer cleft. (F) Residues establishing the strongest interactions with AuTAML are shown as licorice with carbon atoms depicted in light blue. AuTAML is shown with yellow carbon atoms, while the other atoms are colored by atom name. The coordination sphere of the gold atom (represented as a van der Waals sphere) is shown in black dashed lines.

As thiol redox systems are important players in cancer survival, we next investigated the potential interaction of the complexes with key enzymes belonging to these pathways. Purified TrxR1 and GR were incubated with increasing concentrations of the metal complexes or TAML and the enzymatic activities were evaluated spectrophotometrically, as described in the Experimental section. As mentioned above, GR, like thioredoxin reductase, belongs to the family of pyridine nucleotide oxidoreductases but does not bear the thiol/selenol catalytic motif at the C-terminus. As shown in Figure 4A, a strong inhibition of TrxR1 activity was observed at low nanomolar concentrations of the Au(III) complex, while neither the ligand nor CuTAML triggered inhibition of the enzyme. Furthermore, when we compared the action of the three compounds on GR, no decrease of activity could be observed even with AuTAML at ca. 1 μM concentration (Figure 4B). In Figure 4C, the inhibitory effect of AuTAML on the two isoforms of thioredoxin reductase, namely, TrxR1 (cytosolic) and TrxR2 (mitochondrial), is reported. We observed a strong decrease in activity in both isoforms with a major effect on the cytosolic TrxR, in line with previous reports.^69^ As the selenocysteine present in TrxR could be the target of the gold complex, binding of the compounds to TrxR1 was studied by the biotinylated iodoacetamide (BIAM) assay. BIAM is a thiol-tagging reagent, which selectively alkylates the enzyme in a pH-dependent manner: at pH 6.0, only selenocysteines (and low p*K*_a_ cysteines) are alkylated, whereas at pH 8.5, both cysteines (Cys) and selenocysteines (Sec) are covalently modified. In detail, the metal complexes and their free ligand (10 μM) were incubated in the presence of a pre-reduced aliquot of TrxR1. Then, aliquots of the reaction mixture were added to 100 mM BIAM in buffer at either pH 6.0 or pH 8.5 to alkylate the remaining SH/SeH groups, and then analyzed by SDS-PAGE. In this assay, the band intensity is inversely correlated with the ability of the compounds to bind to the selenocysteine (pH 6.0) or cysteines (pH 8.5) present in TrxR1. The immunoblotting (Figure 4D) indicates that only the Au(III) complex is able to bind to the cysteines and the selenocysteines located in the redox active site of TrxR1, while CuTAML or the ligand alone are completely ineffective, in line with the observed lack of inhibition of TrxR1 enzymatic activity. The fact that AuTAML targets cysteine residues as well might explain its slight effect on GR at the highest concentration tested (Figure 4B). This feature is shared with other Au(III) complexes, which are also stronger oxidants than Au(I) compounds.^42^ It should be noted that the nature of the gold/thiolate or gold/selenolate adduct cannot be established yet; in fact, the TrxR inhibition may be due to either Au(III) or Au(I) species, with the latter resulting from intracellular redox speciation of AuTAML. In fact, other Au(III) complexes featuring bidentate N^N ligands have been shown to eventually generate Au(I)-protein adducts.^50,70^

Since AuTAML has been shown to effectively inhibit the enzymatic activity of TrxR1 (Figure 4A,C) and to be able to target the selenocysteine in the enzyme active site (Figure 4D), we also built a model of this compound covalently bound at Sec-498 following substitution of one of the chloride ligands (Figure 4E,F). As a result, the MD simulation of this coordination adduct showed that the ligand can bind at the interface of the two TrxR1 homodimers, establishing a network of stabilizing hydrophobic interactions with L112, S11, and π-stacking interactions with Y116 and H108. As such, by covalently blocking the TrxR’s active site, AuTAML is likely to exert a dual-mode activity, involving both ERα and TrxR.

### Redox Enzyme Activities in Breast Cancer Cells after Treatment with TAML Complexes

**Figure 5:**
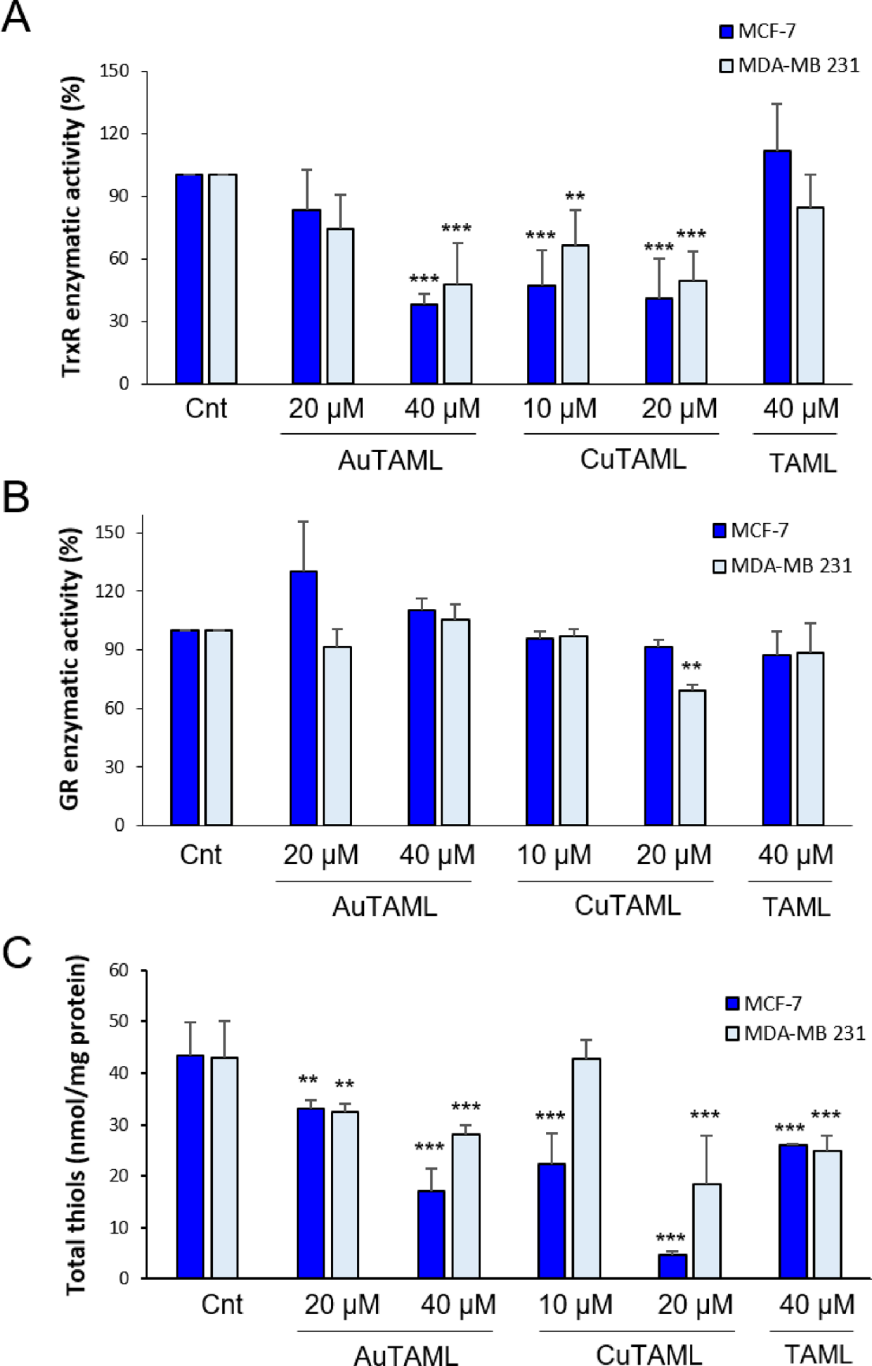
Effect of the metal complexes and their ligand on TrxR and GR activities and total thiol groups. MCF-7 and MDA-MB 231 cells were treated with the complexes or TAML for 48 h, then lysed, and subjected to the estimation of TrxR (A) and GR (B) enzymatic activities as described in the Experimental section. Values are reported as percentage of enzymatic activity relative to the control. The mean values ± SD of 3 experiments are reported. **p < 0.01; ***p < 0.001. (C) Total -SH groups were titrated with DTNB as described in the Experimental section and expressed as nmol/mg protein. Mean values ± SD of 3 experiments are reported. **p < 0.01, ***p < 0.001.

The inhibitory effect of the TAML derivatives on the enzymatic activity of TrxR and GR was tested in MCF-7 and MDA-MB 231 cells treated for 48 h with the compounds (10–40 μM) in comparison to the free ligand (40 μM). It is crucial to point out that this experimental setting differs from the former cytotoxicity assay, as a greater number of cells is necessary to perform these enzymatic analyses. As a consequence, also the concentration of the metal complexes has been augmented (see Experimental section for details). Regarding the obtained results, TrxR activity decreased when cells were treated with AuTAML but not with its ligand TAML (Figure 5A) as expected from the *in vitro* and *in silico* data. An interesting outcome was obtained with CuTAML that was effective in decreasing TrxR activity as well. This result could imply that CuTAML may deplete cells of reducing equivalents eliciting a redox stress, thus leading to an exhaustion of TrxR activity. This hypothesis is supported by the fact that also GR activity was affected in cells treated with CuTAML (Figure 5B). On the contrary, GR activity was not altered by AuTAML and TAML. In order to investigate whether the incubation with TAML complexes induces a redox imbalance in treated cells, we next determined the total cellular thiols. As shown in Figure 5C, the metal complexes promoted thiol oxidation and especially CuTAML, which resulted in it being the most potent. Altogether, these data suggest that the complexes are able to induce oxidative stress to cancer cells, AuTAML via TrxR inhibition, while CuTAML acting as a cysteine oxidizing agent.

### Mitochondrial Functionality in the Presence of TAML Complexes

**Figure 6:**
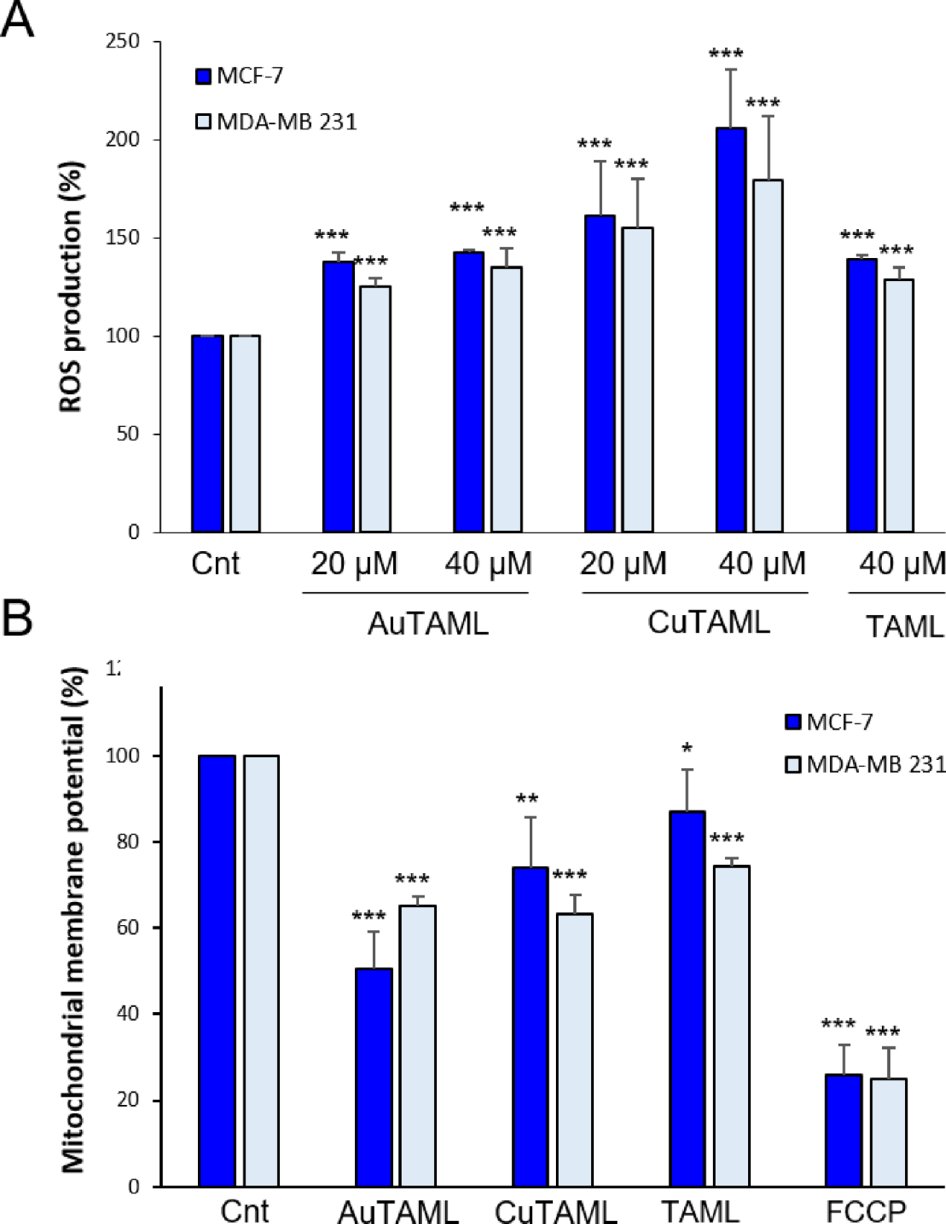
Effect of the compounds on mitochondrial ROS production and on mitochondrial membrane potential. (A) Estimation of ROS production. The two breast cancer cells lines (10^5^/well) were treated with 20 and 40 μM of the metal complexes or TAML for 3 h, and ROS production was evaluated using the DHR probe as described in the Experimental section. Results are reported as the percentage of ROS production compared to the control (Cnt). (B) Assessment of the mitochondrial membrane potential. Cells were treated with 40 μM of the complexes or their ligand for 3 h. Afterward, their mitochondrial membrane potential was determined using the probe tetramethyl rhodamine methyl ester as reported in the Experimental section. The difference in the fluorescence values, representative of the mitochondrial membrane potential, is reported as percentage with respect to untreated cells (Cnt). FCCP (carbonyl cyanide-*p*-trifluoromethoxy phenylhydrazone) was used as reference for mitochondrial membrane potential disruption. Mean values ± SD of 4 experiments are reported for each assay. *p < 0.05, **p < 0.01, ***p < 0.001.

Reactive oxygen species (ROS) are involved in cellular redox homeostasis and are products of physiological mitochondrial metabolism. Their overproduction indicates a perturbation of the cellular antioxidant defense system. In particular, mitochondria can generate superoxide anion, which is rapidly converted into hydrogen peroxide (H_2_O_2_). It has been previously shown that TrxR inhibition by gold compounds causes increases of hydrogen peroxide concentration as well as an imbalance in the intracellular cell redox state leading to mitochondrial membrane permeabilization and swelling.^71,72^ Therefore, the effects on the mitochondrial production of H_2_O_2_ following 3 h incubation with the metal complexes and TAML of both the breast cancer cells were studied. All the compounds were able to significantly increase the basal production of hydrogen peroxide in both cell lines (Figure 6A), with the Cu(II) complex being again the most effective.

Since mitochondria are one of the major ROS producers in the cell, we speculated that they could be targeted by our metal complexes. Mitochondrial membrane potential (ΔΨ_m_) is an important parameter to evaluate the mitochondrial functionality and the loss of ΔΨ_m_ promotes the release of pro-apoptotic factors such as cytochrome c, driving cell death.^72^ Thus, we evaluated the effects of the compounds on ΔΨ_m_ in the two breast cancer cell lines. As depicted in Figure 6B, untreated control showed high fluorescence that indicates high ΔΨ_m_, while the treatment with the metal complexes rapidly decreased it, suggesting that the mitochondrial membrane potential had partially collapsed. In addition, AuTAML and CuTAML showed greater effectiveness in inducing the drop of mitochondrial membrane potential with respect to TAML alone in both cell lines. These data are consistent with their greater capacity of promoting ROS production and imply mitochondrial functional alterations.

**Figure 7:**
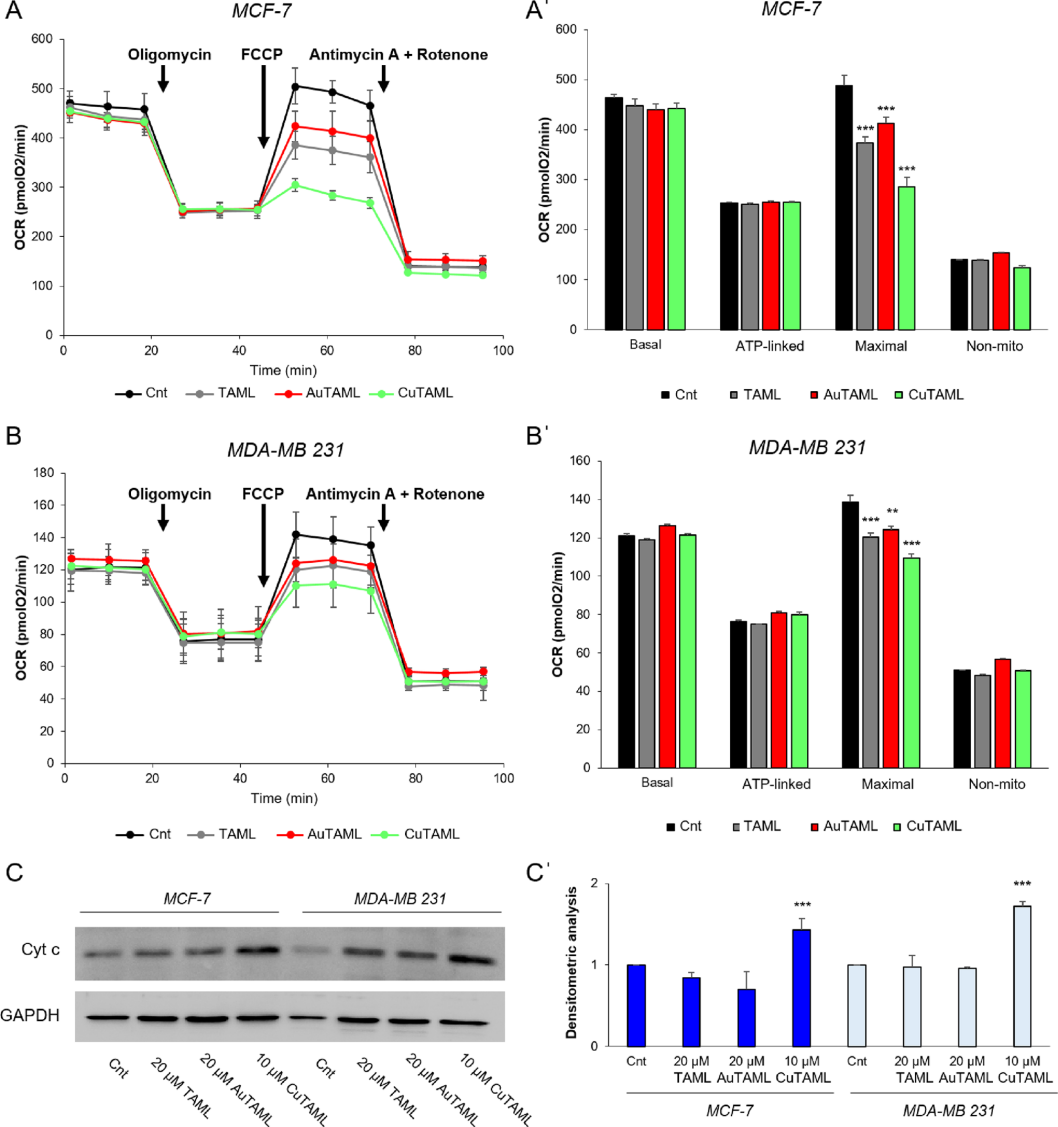
Oxygen consumption rates and western blot analysis of cytochrome c release in cells treated with the complexes or TAML. MCF-7 (A, A′) and MDA-MB 231 (B, B′) cells were treated with 5 μM of the metal complexes or TAML for 3 h. Afterward, the analysis of oxygen consumption rate (OCR) was performed using the Seahorse Xfe24 analyzer as described in the Experimental section. Basal respiration and respiratory capacity in the presence of sequential addition of 1 μM oligomycin (Oligo), 0.5 μM FCCP, and the combination of 1 μM antimycin A + 1 μM rotenone were performed. Mean ± SD of 3 experiments is shown. **p < 0.01, ***p < 0.001. (C, C′) Cells were treated with the complexes or TAML at the indicated concentrations for 18 h and then subjected to determination of mitochondrial cytochrome c release as reported in the Experimental section. (C) Western blot relative to cytochrome c and GAPDH as a loading control. (C′) Densitometric analysis of cytochrome c bands normalized on GAPDH. Mean values ± SD of 3 experiments are reported. ***p < 0.001.

To better characterize the mechanism of action of TAML compounds on mitochondrial functioning, the effects of the metal complexes and TAML ligand on the oxygen consumption rate (OCR) and the extracellular acidification rate (ECAR) of live cells were analyzed using a Seahorse analyzer. With this approach, key cellular functions such as mitochondrial respiration and glycolysis can be determined at the same time in living cells treated with the different metal complexes in a multiwell plate (see Experimental section for details and Figure S14). The OCR was assessed after cell treatment with 5 μM of the metal complexes or TAML for 3 h and with the subsequent addition of different molecules affecting the respiratory chain and ATP production, enabling the determination of the basal, ATP linked, maximal and non-mitochondrial respiration – namely, (i) oligomycin A, a complex V inhibitor, (ii) FCCP, an uncoupling agent, (iii) rotenone, a complex I inhibitor, and (iv) antimycin A, a complex III inhibitor (see also explanatory Figure S14). In Figure 7A,A′, the results obtained on MCF-7 cells show that all the three compounds significantly reduced the mitochondrial maximal respiration capacity without changing the basal and the ATP-linked respiration rates. In fact, basal respiration rates were similar, and oligomycin A decreased OCR equally effectively in control and treated cells, indicating no changes in ATP-linked respiration and showing that the complexes do not have an uncoupling effect. The differences appeared only when FCCP was added, leading to the maximal velocity of oxygen consumption. In this condition, the treated cells were less able to increase their respiration rate with respect to the control. The addition of rotenone and antimycin A finally blocked the electron flow across the respiratory chain and led to a level of oxygen consumption similar among the treatment groups, suggesting no difference in non-mitochondrial OCR.

Interestingly, CuTAML was the most effective in decreasing the maximal respiration in line with its greater capacity of inducing ROS production and decrease of the mitochondrial membrane potential (Figure 6). AuTAML was also able to decrease cellular maximal respiration, but with a potency similar to the ligand, suggesting that the TAML scaffold has *per se* an inhibitory activity on mitochondrial respiration. A similar scenario was evident in MDA-MB 231 cells (Figure 7B,B′) where the maximal respiratory capacity was also singularly affected. Of note, in the ER^–^ breast cancer cell line, the difference in potency on the maximal respiration between the compounds is less marked than in MCF-7 cells, but again CuTAML appears to be the most potent, while AuTAML and TAML have a similar performance on mitochondrial impairment. Overall, these data suggest that the new complexes are able to affect the mitochondrial oxygen consumption and thus the bioenergetic functions of the cancer cells, especially influencing the maximal respiration.

It should be noted that the basal respiration involves a fraction of the total mitochondrial bioenergetic capability while, when the energy requirement increases, the mitochondrial respiration can rapidly rise to the maximum level to enable energetic adaptation and synthesize more ATP. Likewise, the treatment with FCCP leads to the maximal oxygen consumption, as mitochondria are forced to continuously restore the proton gradient dissipated by the uncoupler, artificially mimicking energy needs. The difference between basal and maximal respirations represents the mitochondrial reserve capacity (RC).

RC is a readout of mitochondrial fitness, and a lower RC corresponds to mitochondrial dysfunction. As a consequence, cells unable to increase their oxygen consumption rates are more prone to undergoing an energetic defect and therefore are at risk of cell death.

RC is regulated by several signals coming from inside and outside the mitochondria, and oxidative stress was previously shown to reduce it.^73^ In our model, the treatment of breast cancer cells with TAML compounds impacts their RC, indicating mitochondrial damage, which was particularly evident upon treatment with CuTAML. Thus, the fact that an increased ROS production was observed, suggests the involvement of oxidative stress in the decreased RC.

Afterward, we investigated whether the observed impairment in the respiration associated with the decrease of the mitochondrial membrane potential could lead to the activation of the apoptotic pathway. In fact, it is known that the decrease of the mitochondrial membrane potential is an upstream event to the release of cytochrome c from the mitochondrial intermembrane space.^74^ Eventually, cytosolic cytochrome c induces the assembly of the apoptosome that is required for activating downstream caspases.^75^

Thus, with the aim of dissecting the type of cell death pathway activated by our new complexes, we checked for cytochrome c release (Figure 7C,C′). AuTAML and TAML led to a similar outcome, with a mild effect on cytochrome c release after 18 h treatment. This result is in line with the low effect on mitochondrial respiration observed in the treated cells (Figure 7A,B). Conversely, CuTAML was again the most potent and rapid complex and induced a massive release of cytochrome c from mitochondria to the cytosolic compartment, eventually promoting the cell death. These observations further validate the mechanism through which the complexes exert their high cytotoxic effect acting as mitochondrial disruptors and triggering apoptotic pathway activation. Of note, also the parent compound tamoxifen was reported to induce apoptosis activation, indicating a common outcome due to the backbone structure able to target mitochondria.^76^

We then wanted to explore whether the observed impairment in the OCR and mitochondrial activity could be also due to an upstream effect of the complexes on cellular catabolic pathways. Since glycolysis is the principal pathway fueling the Krebs cycle and mitochondrial functions, we determined the glycolytic activity by measuring the extracellular acidification rate (ECAR) of live cells through the Seahorse analyzer. The results reported in Figure S15 of the SI demonstrate that the glycolytic flux is not impaired by cell treatment with the TAML compounds. Altogether, these data indicate that the complexes affect oxidative phosphorylation, but not glycolysis, to exert their cytotoxic activity against cancer cells, suggesting a targeted action on mitochondria.

**Figure 8:**
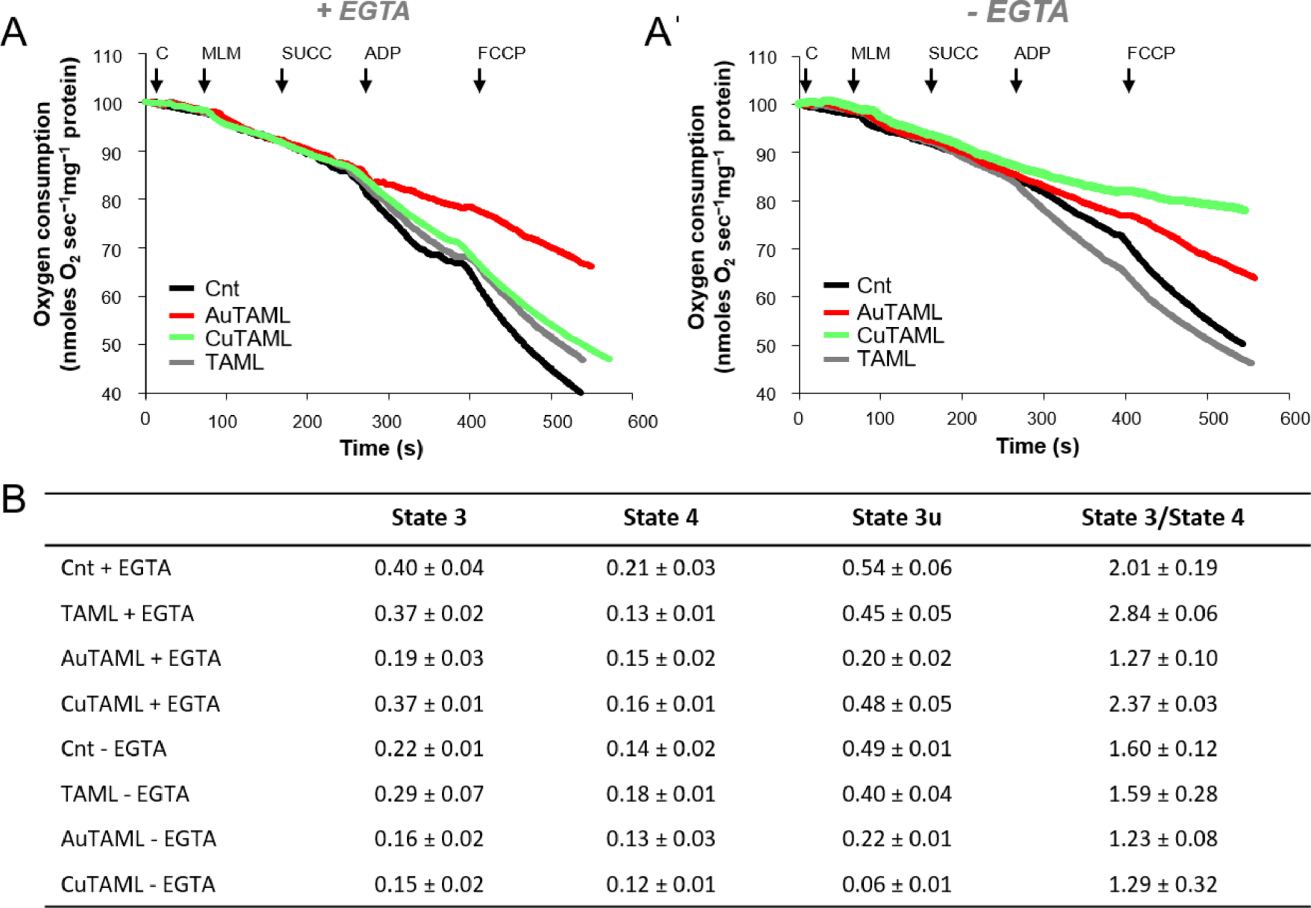
Mitochondrial respiratory capacity of isolated mouse liver mitochondria in the presence of the TAML compounds. After mitochondrial isolation, oxygen consumption was measured using succinate (SUCC) as the substrate, in the presence or absence of EGTA, employing a Clark electrode (see the Experimental section for details). Representative curves relative to the oxygen consumption in the presence (A) or absence (A′) of EGTA are shown. C = 5 μM compound; MLM = mouse liver mitochondria. (B) Table reporting the rates of oxygen consumption in each state: state 3 (ADP addition), state 4 (succinate addition, SUCC), and state 3u (FCCP addition). The respiratory control index (state 3/state 4) is also reported. Mean ± SD of 3 experiments is shown.

To shed further light into the mode of action of the three compounds, we tested them directly on the isolated mouse liver mitochondria (MLM) analyzing mitochondrial respiration using a Clark electrode (see the Experimental section for details) observing interesting differences in the mechanism of action of the TAML metal complexes. As reported in Figure 8, in the presence of a divalent cation chelator (EGTA), the only compound impairing mitochondrial oxygen consumption was AuTAML, whereas TAML and CuTAML were almost completely ineffective. Performing the same analysis in the absence of EGTA led to a different outcome. Indeed, AuTAML had a similar effect on mitochondrial function to that in the presence of EGTA, while CuTAML became the most active inhibitor of mitochondrial respiration. Of note, it is possible to observe that TAML is ineffective, both in the presence or absence of EGTA. This data reveals that although both metal complexes induce a mitochondrial impairment in the oxygen consumption of cancer cells, their mechanisms of action are different and dependent on the metal reactivity. In particular, CuTAML does not act directly on the electron transport chain as AuTAML but induces most likely mitochondrial swelling.

**Figure 9:**
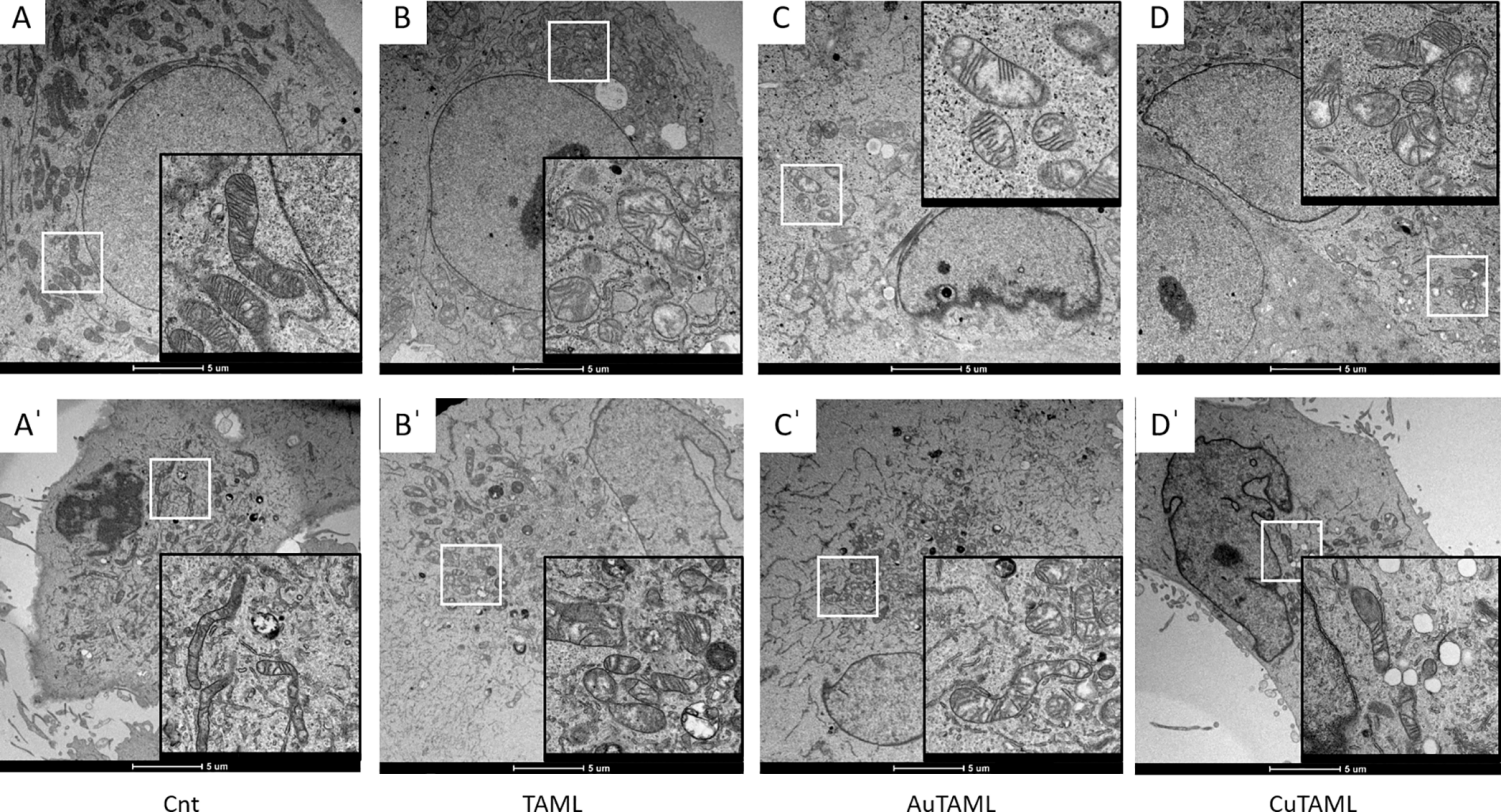
TEM analysis of mitochondrial morphology in cells treated with the novel compounds. MCF-7 (upper panels) and MDA-MB 231 (lower panels) cells were treated with 10 μM AuTAML and TAML or with 5 μM CuTAML for 3 h. Afterward, cells were fixed, subjected to inclusion, and observed at the TEM as reported in the Experimental section. Representative microphotographs of cells and mitochondria for each treatment are reported. (A, A′) Cnt; (B, B′) 10 μM TAML; (C, C′) 10 μM AuTAML; (D, D′) 5 μM CuTAML; details are magnified in the insets.

Considering all the functional alterations observed in mitochondria, we finally decided to look at their morphology in treated cancer cells by transmission electron microscopy (TEM). TEM analysis shows that the untreated control for both MCF-7 and MDA-MB 231 cells presents mitochondria in large number and homogeneously distributed throughout the cytoplasm (Figure 9A,A′). In cells treated with TAML (10 μM, Figure 9B,B′), an early damage was evident. However, following treatment with AuTAML (10 μM, Figure 9C,C′), the mitochondrial matrix is also rarefied, with a de-arrangement of the cristae (see inset) and mitochondrial swelling. CuTAML-treated cells also showed mitochondrial swelling and changes in mitochondria morphology, especially in MCF-7, already at 5 μM concentration (Figure 9D,D′). The decrease of oxygen consumption observed in the absence of EGTA when respiration was assessed in isolated mitochondria (Figure 8) can be referred to a swelling effect, which is confirmed by TEM analysis of mitochondria in the cell environment. In CuTAML-treated cells, it is also possible to observe a large number of cytosolic vacuoles as previously reported for other copper complexes.^77^ This sequence of ultrastructural changes is in agreement with our finding regarding the impairment of mitochondrial function, such as the abovementioned decrease of the mitochondrial membrane potential, the alteration of the respiratory chain capacity (OCR), and the imbalance of respiration in isolated mouse mitochondria.

## Conclusions

**Figure 10:**
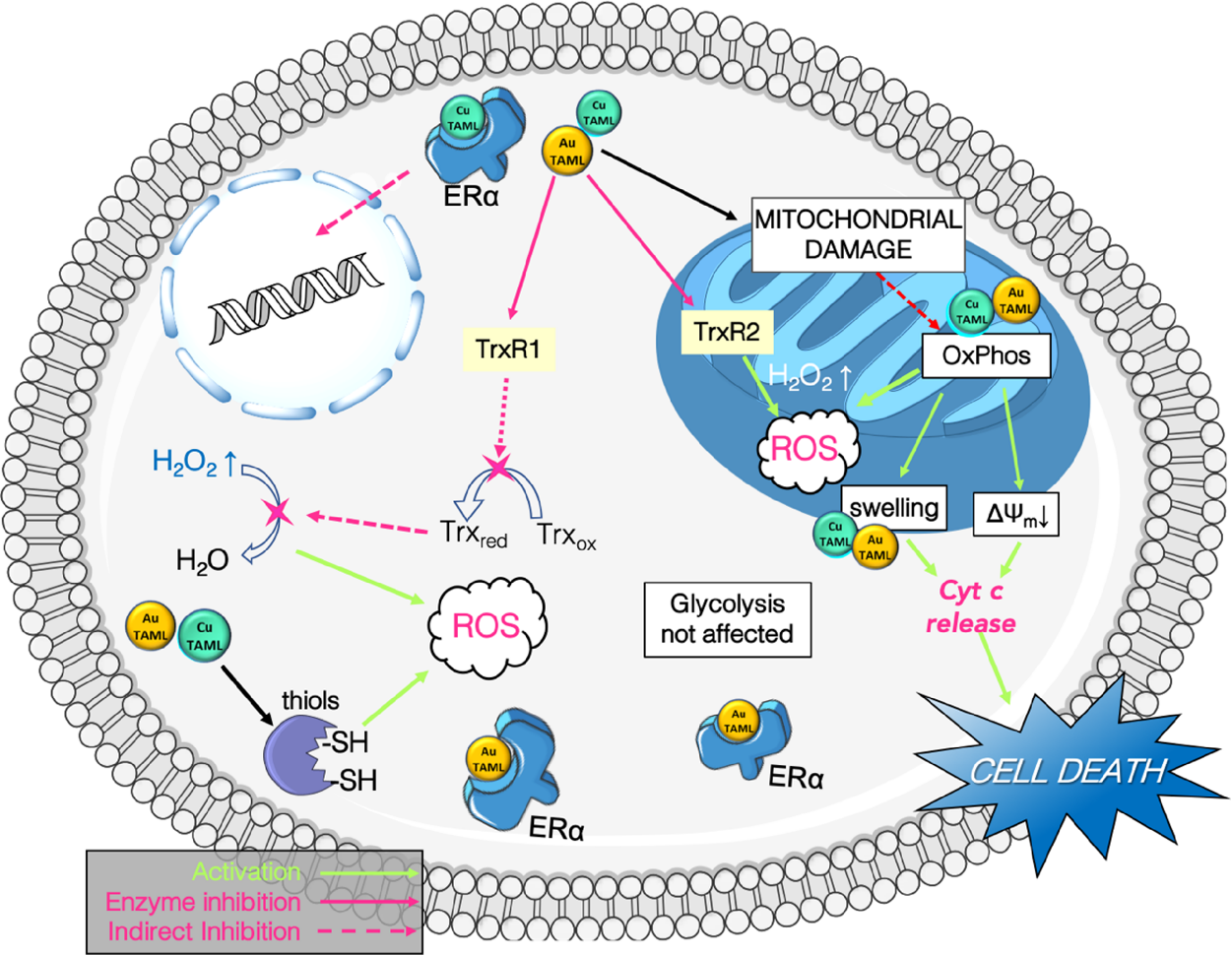
Infographic representation of the proposed mechanism of action of Au(III) and Cu(II) TAML complexes in breast cancer cells.

In summary, we report the synthesis, characterization, and anticancer potential of two ‘hybrid’ metallodrugs combining the bioactive properties of the metal centers with the ERα targeting capability of a tamoxifen-type ligand. The proposed mechanism of action of the gold and copper TAML complexes is reported in Figure 10.

*In vitro*, the metal complexes and their ligand showed marked anti proliferative activity especially in ER^+^ MCF-7 cells, suggesting that they retain the ability to interact with the ERα as the active metabolite of tamoxifen, namely, 4-hydroxytamoxifen. Indeed, MD simulation studies on the interactions of the two compounds with the ERα LBD support this hypothesis. The two metallodrugs were also able to induce a redox imbalance in breast cancer cells (both ER^+^ and ER^–^), as shown by an increased ROS production and reduction of thiol groups. Furthermore, the Au(III) derivative AuTAML showed potent inhibition of thioredoxin reductase activity, occurring via the metal coordination to the selenocysteine present in the active site of the enzyme, as shown by *in vitro* and *in silico* studies. Instead, in line with previous observations,^78,79^ the Cu(II) complex exerts its cytotoxic effect as a pro-oxidizing agent, triggering ROS production and inducing a general oxidation of cysteine residues in different protein targets. It is worth mentioning that several Cu(II) compounds with N^N donor ligands have already been reported for their anticancer properties and hypothesized to act via a multimodal spectrum of biological activity, including apoptosis induction by a mitochondrial pathway.^79−81^ Interestingly, Cu(II)-phenanthroline complexes have also been recently described as inhibitors of the aquaporins,^82^ membrane water and glycerol channels often abnormally expressed in tumors of different origins,^83^ suggesting an additional mechanism of action for this family of metallodrugs.

Of note, our results show that the metal TAML compounds can target mitochondria via different mechanisms, leading to a decrease of the mitochondrial membrane potential. The TAML ligand itself can affect mitochondria, inducing a decrease of the maximal respiration and reserve capacity, pointing out that the common ligand scaffold is also responsible for the mitochondrial damage, and corroborating the value of the ‘hybrid’ metallodrug concept. The mitochondrial targeting activity of the compounds leads to cytochrome c release, triggering activation of apoptosis, with the Cu(II) complex being the most effective.

The obtained results from the OCR and TEM analysis point toward important differences between the two metal TAML complexes. For example, CuTAML was the most effective in decreasing the maximal respiration in line with its greater capacity of inducing ROS production and decrease of the mitochondrial membrane potential. Moreover, the Cu(II) complex was the most effective in inducing mitochondrial swelling and promoting the release of pro-apoptotic factors, likely due to extensive oxidative damage. Overall, these effects led to the higher antiproliferative activity observed for CuTAML in both cancer cell lines with respect to AuTAML and TAML. Considering the crucial role of copper in mitochondrial function and metabolism,^84^ it is not surprising that the alteration of its homeostasis is particularly detrimental in these organelles. However, the effects of ROS production and depletion of intracellular free thiols could also account for the pronounced cytotoxicity of CuTAML.

In the case of AuTAML, the observed inhibition of mitochondrial processes is in line with previous studies on Au(III) and Au(I) complexes,^18,85^ although no effects on glycolysis could be observed in this case. In addition to TrxR inhibition, AuTAML exerted its action by interfering with the electron transport chain electron flux, as suggested for example by the evaluation of the mitochondrial respiratory capacity in isolated mouse liver mitochondria, thus featuring a more defined pathway of mitochondrial damage and overall cytotoxicity than CuTAML.

In conclusion, the novel TAML derivatives display anticancer activity toward both estrogen-sensitive and insensitive breast cancer cells by inducing oxidative stress and promoting mitochondrial dysfunctions overcoming the cancer resistance. This research constitutes a starting point for further studies on this new family of hybrid metallodrugs, which can overcome cancer resistance.

## Experimental Section

### General

Solvents and reagents (reagent grade) were all commercially available and used without further purification. Reactions involving dry solvents were performed under a nitrogen atmosphere and in flame-dried glassware, while syntheses involving Au(III) or Cu(II) were carried out in the dark. The remaining manipulations, unless otherwise stated, were conducted under standard conditions. Flash column chromatography was performed on a Biotage Isolera Four supported with the due cartridges. NMR spectra were recorded on a Bruker AVANCE DPX 400 at room temperature (400.13 MHz for ^1^H NMR, 100.53 MHz for ^13^C[^1^H] NMR). Chemical shifts δ are reported in parts per million (ppm) with respect to residual ^1^H and ^13^C signals of the deuterated solvents as the internal standard, whereas coupling constants *J* are denoted in Hertz (Hz). The following abbreviations as well as combinations thereof are employed for NMR signal multiplicities: s = singlet, d = doublet, t = triplet, p = quintet. Elemental analyses (EAs) for C, H, N, and S were obtained from a HEKAtech Euro EA by the microanalytical laboratory at the Technical University of Munich. The purity of both complexes (AuTAML and CuTAML) was determined to be >95% by EA.

HR-ESI-MS was conducted on a Thermo Fisher Exactive Plus Orbitrap mass spectrometer, which was equipped with an ESI source by the same company. Samples were prepared in MeCN, syringe-filtered to 0.45 μm, and directly injected. With an ionization voltage of 2 kV, ions were detected in the positive mode.

### Synthesis and Characterization

TAML and its intermediates were prepared in a 4-steps synthesis (Scheme S1) as reported by Hey-Hawkins and coworkers.^37^ To afford highly pure compounds, the eluent mixtures for the column chromatography were modified accordingly.

#### AuTAML

45 mg of TAML (0.11 mmol, 1.00 equiv) was dissolved in a round-bottom flask using 3 mL of MeCN. To the resulting yellow solution, under vigorous stirring, 8 mL of sodium tetrachloroaurate(III) dihydrate (44.5 mg, 0.11 mmol, 1.00 equiv) in biograde H_2_O was added, causing a color shift to orange and the appearance of a dark precipitate. After the addition of an excess of KPF_6_ (59 mg, 320 μmol, 3.00 equiv), the reaction mixture was refluxed for 4 h protected from light, during which a dark orange clear solution was achieved. Upon cooling to room temperature, the reaction mixture was filtered, and the desired product collected as a dark orange solid (69.8 mg, 0.84 mmol, 78%). Further purification was achieved by re-dissolving the compound in MeCN and filtering off the solution.

^1^H NMR (400 MHz, CD_3_CN) δ 9.40 (d, *J* = 6.1 Hz, 1H), 9.05 (d, *J* = 6.4 Hz, 1H), 8.52 (t, *J* = 7.9 Hz, 1H), 8.26 (d, *J* = 8.1 Hz, 1H), 8.20 (s, 1H), 8.00 (t, *J* = 7.6 Hz, 1H), 7.67 (dd, *J* = 6.5, 2.1 Hz, 1H), 7.23 (d, *J* = 8.7 Hz, 2H), 6.99 (d, *J* = 8.7 Hz, 2H), 6.91 (d, *J* = 8.8 Hz, 2H), 6.70 (d, *J* = 8.7 Hz, 2H), 3.83 (s, 3H), 3.66 (s, 3H), 2.71 (q, *J* = 7.5 Hz, 2H), 1.02 (t, *J* = 7.4 Hz, 3H).

^13^C NMR (101 MHz, CD_3_CN) δ 163.01 (ar), 160.67 (ar), 160.37 (ar), 156.68 (ar), 155.44 (ar), 148.57(ar), 147.51 (ar), 147.25 (ar), 146.73 (ar), 137.22 (ar), 135.00 (ar), 134.53 (ar), 133.58 (ar), 131.53 (ar), 131.00 (ar), 130.64 (ar), 128.75 (ar), 127.00 (ar), 114.78 (*C=C*), 114.74 (*C=C*), 56.05 (CH_2_), 28.46 (CH_3_), 13.72 (CH_3_).

HR-ESI-MS (CH_3_CN, pos. mode) for C_28_H_26_AuCl_2_N_2_O_2_^+^(ML^+^): exp. 689.1013 (calc. 689.10314).

EA, calculated for C_28_H_28_AuCl_2_F_6_N_2_O_3_P (**AuTAML**·H_2_O) [%]: C, 39.41; H, 3.31; N, 3.28. Found [%]: C, 39.48; H 3.04; N, 3.41.

#### CuTAML

A 12 mL solution of dry CuCl_2_ (16.1 mg, 0.12 mmol, 1.00 equiv) in dry DCM was added portion-wise to a flame dried two-neck round-bottom flask equipped with 50 mg of TAML (0.12 mmol, 1.00 equiv). The dark green solution was left stirring at room temperature overnight under light exclusion, prior to the solvent evaporation by reduced pressure. The resulting solid was redissolved in the minimum amount of MeCN prior to the addition of an excess of diethylether. The final complex was collected as a dark green powder by centrifuging the suspension and washing it with 3 further aliquots of diethylether (32 mg, 0.58 mmol, 50%).

HR-ESI-MS (CH_3_CN, pos. mode) for C_28_H_26_ClCuN_2_O_2_^+^(ML-Cl^+^): exp. 520.0957 (calc. 520.09733).

EA, calculated for C_28_H_26_Cl_2_CuN_2_O_2_ (**CuTAML**) [%]: C, 60.38; H, 4.71; N, 5.03. Found [%]: C, 60.21; H 4.71; N, 5.13.

### UV–Visible Stability Studies

UV–visible absorption spectra to investigate the complexes stability in solution were recorded on a Cary 60 UV–Vis spectrometer (Agilent Technologies, Santa Clara, USA). Stock solutions (2 mM) in MeCN were prepared prior to their dilution to 60.0 μM in 1× PBS (pH 7.4). UV–vis spectra were collected at room temperature and at 37 °C, over 24 h at different intervals (every 15 min during the first hour and 30 min for the remaining 23 h). The same conditions were applied to study the stability of AuTAML in the presence of 2 equiv GSH at 37 ° C, over 24 h. Data visualization and analysis were performed using Origin (OriginLab Corporation, Northampton, MA, USA).

### X-ray Crystallography

Crystals of AuTAML were obtained by slow evaporation of a saturated solution of MeCN. X-ray intensity data were measured on a Bruker D8 Venture single crystal X-ray diffractometer equipped with a CMOS detector (Bruker Photon-100), a TXS rotating anode with Mo Kα (λ = 0.71073 Å), and a Helios mirror optic using the software package APEX4.^86^ Measurements were performed on a single crystal coated with perfluorinated ether, and then the crystal was fixed on top of a Kapton microsampler. This was then transferred to the diffractometer and frozen under a stream of cold nitrogen. A matrix scan was used to determine the initial lattice parameters. Reflections were corrected for Lorentz and polarization effects, scan speed, and background using SAINT.^87^ Absorption correction, including odd and even spherical harmonics, was performed using SADABS.^88^ Space group assignments were based upon systematic absences, E statistics, and successful refinements of the structure. The structure was solved by direct methods using successive difference Fourier maps, refined using APEX III software, in conjugation with SHELXL-2014/5 and SHELXLE.^89,90^ Non-hydrogen atoms were refined with anisotropic displacement parameters. Full-matrix least-squares refinements were carried out by minimizing ∑*w*(*F*_o_^2^ – *F*_c_^2^)^2^ with the SHELXL weighing scheme.^87,88^ Neutral atom scattering factors for all atoms and anomalous dispersion corrections for the non-hydrogen atoms were obtained from International Tables for Crystallography.^91^ Images of the crystal structure were generated using Mercury.^92^ The CCDC code is 2253648. Authors will release the atomic coordinates upon article publication.

### Biological Assays

#### Cell Culture

The breast cancer cell models MCF-7 (ER^+^) and MDA-MB 231 (ER^–^) were grown in adhesion at 37 °C in a 5% carbon dioxide atmosphere, using high-glucose Dulbecco’s modified Eagle’s medium (DMEM) containing glutaMAX and supplemented with 10% fetal calf serum and Pen-Strep (Thermo Fisher Scientific, Waltham, MA, USA).

#### MTT Assay

Cell viability was determined with the 3-[4,5-dimethylthiazol-2-yl]-2,5-diphenyltetrazolium bromide (MTT) reduction assay. Cells (1 × 10^4^ cells/well) were plated in a 96 well plate and the next day treated with increasing concentrations of the metal complexes or the ligand TAML for 24 or 48 h. Stock solutions of the compounds (10 mM) were freshly prepared in MeCN prior the experiment. At the end of the treatment, cells were incubated for 3 h at 37 °C with 0.5 mg/mL MTT (Sigma-Aldrich, St. Louis, MO, USA) dissolved in phosphate-buffered saline (PBS). Afterward, 100 μL of stop solution (90% isopropanol/10% dimethyl sulfoxide) was added to each well. After 15 min, the absorbance at 595 and 690 nm was estimated using a Tecan Infinite M200 PRO plate reader (Tecan, Mannedorf, CH).

#### Estimation of the Inhibitory Effect of the New Complexes on the Enzymatic Activity of Isolated TrxR1, TrxR2, and GR

Highly purified cytosolic (TrxR1) and mitochondrial (TrxR2) thioredoxin reductases were prepared from rat liver according to Luthman and Holmgren^93^ and Rigobello and Bindoli,^94^ respectively. The protein content of the purified enzyme preparations was measured according to Lowry *et al*.^95^ Glutathione reductase from baker’s yeast was purchased from Sigma-Aldrich (St. Louis, MO, USA). Thioredoxin reductase activity was determined by estimating the DTNB reducing properties of the enzymes in the presence of NADPH. Aliquots of highly purified TrxR in 0.2 M Na–K–Pi buffer (pH 7.4) added to 5 mM EDTA were pre-reduced with 0.25 mM NADPH. Then, the compounds were added at different concentrations and the reaction was initiated with 1 mM DTNB and monitored spectrophotometrically at 412 nm for about 10 min on a Lambda 2 spectrophotometer (PerkinElmer, Waltham, MA, USA). GR activity was measured in 0.2 M Tris–HCl buffer (pH 8.1), 1 mM EDTA, and 0.25 mM NADPH in the presence of the compounds at increasing concentrations. The assay was started by the addition of 1 mM GSSG and followed spectrophotometrically at 340 nm.

#### BIAM Assay

Isolated rat liver TrxR1, pre-reduced in the presence of 60 μM NADPH, was incubated with 10 μM of the metal complexes or the ligand for 30 min at room temperature, in 50 mM Tris–HCl buffer (pH 7.4) containing 0.2 mM NADPH and 1 mM EDTA. After incubation, 8 μL of the reaction mixture was added to 8 μL of 100 mM biotinylated iodoacetamide (BIAM) in 0.1 M Tris–HCl at pH 8.5 or in 0.1 M Hepes-Tris pH 6.0.^96^ The samples were incubated at room temperature for an additional 30 min to allow BIAM alkylation of free −SH/SeH groups of the enzyme. Then, samples were subjected to sodium dodecyl sulfate-polyacrylamide gel electrophoresis (SDS-PAGE) on a 10% gel, and transferred to a nitrocellulose membrane. The BIAM-labeled enzyme was detected with horseradish peroxidase-conjugated streptavidin. The detection was performed using a UVITEC Alliance Q9 mini chemiluminescence imaging detector (UVITEC, Cambridge, UK) and analyzed via NineAlliance software for band quantification.

#### TrxR and GR Activities in Cell Lysates

Breast cancer cells (1 × 10^6^) were treated with the tamoxifen derivatives for 48 h. Afterward, cells were harvested and washed with PBS. Each sample was lysed with a modified radioimmunoprecipitation assay (RIPA) buffer composed of 150 mM NaCl, 50 mm Tris/HCl, 1 mM EDTA, 1% Triton X-100, 0.1% SDS, 0.5% sodium deoxycholate, 1 mM NaF, and 0.1 mM phenylmethylsulfonyl fluoride (PMSF) containing an antiprotease cocktail (Complete, Roche, Mannheim, DE). After 40 min at 4 °C, the lysates were centrifuged at 15800*g* to discard the debris and tested for total TrxR and GR activities. For TrxR activity, 50 μg of proteins from cell lysates was tested in 0.2 M Na–K–Pi buffer (pH 7.4) containing 5 mM EDTA and 20 mM DTNB. After 2 min, 0.25 mM NADPH was added and the reaction was followed for about 10 min at 412 nm, 25 °C. GR activity of cell lysates (80 μg of proteins) was measured in 0.2 M Tris/HCl buffer (pH 8.1) containing 1 mM EDTA and 0.25 mM NADPH. The assay was initiated by addition of 1 mM glutathione disulfide and monitored spectrophotometrically at 340 nm, 25 °C.

#### Estimation of Total Thiol Groups

Total cellular thiol groups were measured with Ellman’s assay.^97^ Briefly, cells (3 × 10^5^ cells/well) were treated with the complexes or the ligand for 48 h, then washed with PBS, and lysed with 1 mL of ice-cold 0.2 M Tris–HCl buffer (pH 8.1), containing 7.2 M guanidine. The titration of free thiols after addition of 30 mM 5,50-dithiobis(2-nitrobenzoic acid) (DTNB) was monitored spectrophotometrically at 412 nm. The obtained values were normalized on the protein content measured according to Bradford.^98^

#### Production of Reactive Oxygen Species

MCF-7 and MDA-MB 231 cells (1 × 10^4^ per well) were seeded into a 96 well culture plate in complete growth medium. After 24 h, the cells were treated with the complexes or the ligand in the same medium for 3 h. After a wash with PBS, 1.5 μM of the probe dihydrorhodamine 123 (DHR) (Sigma-Aldrich, St. Louis, MO, USA) dissolved in PBS-glucose (10 mM) was added to the cells and the fluorescence increase was monitored at λ_ex_ = 485 nm and λ_em_ = 527 nm using a plate reader (Tecan Infinite M200 PRO, Männedorf, CH).

#### Measurement of Mitochondrial Membrane Potential

Mitochondrial membrane potential was analyzed using the probe tetramethyl rhodamine methyl ester (TMRM). Cells (2.5 × 10^5^ cells/well) were plated in a 96-well plate and, the next day, treated for 3 h with the complexes in complete growth medium. Afterward, cells were washed in PBS/10 mM glucose and loaded with 0.1 μM TMRM (Molecular Probes, Thermo Fisher Scientific) diluted in PBS/10 mM glucose. Cells were incubated at 37 °C in the dark, for 45 min. Finally, cells were washed twice with 100 μL/well of PBS/10 mM glucose and the mitochondrial membrane potential was estimated at λ_ex_ = 548 nm and λ_em_ = 590 nm on a plate reader (Tecan Infinite M200 PRO, Männedorf, CH). 1.5 μM carbonyl cyanide-*p*-trifluoromethoxy phenylhydrazone (FCCP) was used as a positive control.

#### Analysis of the Mitochondrial Morphology in Treated Cells

Cells were seeded at a density of 5 × 10^5^ cells/well in a 24 well-plate and grown overnight. The next day, cells were treated with 10 μM AuTAML or TAML or 5 μM CuTAML for 3 h. Then, cells were fixed with 2.5% glutaraldehyde in 0.1 M sodium cacodylate buffer pH 7.4 overnight at 4 °C. The samples was postfixed with 1% osmium tetroxide plus potassium ferrocyanide 1% in 0.1 M sodium cacodylate buffer for 1 h at 4 °C. After three water washes, samples were dehydrated in a graded ethanol series and embedded in an epoxy resin (Sigma-Aldrich). Ultrathin sections (60–70 nm) were obtained with an Ultratome Leica Ultracut EM UC7 ultramicrotome, counterstained with uranyl acetate and lead citrate, and viewed with a Tecnai G^2^ (FEI) transmission electron microscope operating at 100 kV. Images were captured with a Veleta (Olympus Soft Imaging System) digital camera.

#### Determination of the Oxygen Consumption of Isolated Mitochondria

Mouse liver mitochondria were isolated by differential centrifugation following the method of Myers and Slater.^99^ Mice were sacrificed by cervical dislocation, and the liver was explanted and dipped in ice-cold isolation buffer (220 mM sucrose, 70 mM mannitol, 0.1 mM EDTA, 5 mM Hepes-Tris (pH 7.0)). Once blood traces were removed, the organ was minced and homogenized. Afterward, the homogenates were centrifuged at 700*g* for 10 min at 4 °C and the obtained supernatants were centrifuged at 10000*g* for 15 min at 4 °C. The pellets (mitochondrial fractions) were suspended and centrifuged at 10000*g* for 15 min at 4 °C in the isolation buffer without EDTA. Concentrations of mitochondrial proteins were estimated with the Bradford method.^98^ Oxygen consumption of mouse liver mitochondria was measured polarographically, utilizing a Clark-type oxygen electrode inserted in a water-jacketed chamber at 27 °C, with constant stirring. Mouse liver mitochondria (1 mg/mL) were incubated in 20 mM Hepes-Tris buffer (pH 7.4), 100 mM sucrose, 50 mM KCl, 1 mM MgCl_2_, and 1 mM NaH_2_PO_4_ with or without the addition of 1 mM EGTA. Then, compounds were added at the final concentration of 5 μM. Respiration was started by the addition of either 7.5 mM succinate or the combination 7.5 mM glutamate + 3.75 mM malate. Then, 0.2 mM ADP was added (state 3). Finally, 0.25 μM trifluoromethoxy carbonylcyanide phenylhydrazone (FCCP) was added to measure the maximal respiration rate (state 3u). The oxygen consumption was calculated in nmol of O_2_·s^–1^ mg^–1^ protein.

#### Analysis of the Mitochondrial Respiration in Treated Cells

Cellular respiration was determined with the Seahorse XFe24 Analyzer (Agilent Technologies, Santa Clara, CA, USA) following the Cell Mito Stress Test protocol. Cells were seeded at a density of 4 × 10^5^ cells/well and grown overnight in complete growth medium. Afterward, cells were treated with the complexes or the ligand for 3 h in the same medium. Before the start of the experiment, the medium was replaced with an XF DMEM Assay Medium (pH 7.4), supplemented with 10 mM glucose, 1 mM sodium pyruvate, and 2 mM glutamine and the cells were subjected to the oxygen consumption analysis at 37 °C. In particular, three measurements were performed of the basal respiration, and after the sequential injections of 1 μM oligomycin, 0.5 μM FCCP, and the combination 1 μM antimycin A + 1 μM rotenone with 2 min of mixing in between measurements.

#### Estimation of Cytochrome c Release

After 18 h of incubation in the presence of the compounds, cells (2 × 10^6^) were harvested, washed with PBS, and treated with a hypotonic lysis buffer formed by 20 mM Hepes-Tris buffer (pH 7.5), 10 mM KCl, 1.5 mM MgCl_2_, 1 mM EDTA, 1 mM EGTA, and antiproteases (Complete, Roche, Mannheim, DE) for 15 min. Then, the suspension was centrifuged at 15800*g* for 10 min at 4 °C and 0.5 mM EGTA and 2.5 mM PMSF were added to the supernatants. A further centrifugation for 30 min at 100000*g* at 4 °C led to the cytosolic fraction. Aliquots of 10 μg of proteins were subjected to SDS-PAGE (15%) followed by western blotting using a cytochrome c monoclonal antibody clone 7H8.C12 (Sigma-Aldrich, St. Louis, MO, USA). A peroxidase conjugated secondary antibody was employed to detect the immunoreactive bands using a UVITEC Alliance Q9 mini chemiluminescence imaging detector (UVITEC, Cambridge, UK).

#### Glycolysis Stress Test Assay

MCF-7 and MDA-MB 231 cells were seeded at a density of 4 × 10^5^ cells/well and grown overnight in complete growth medium. Then, cells were treated with the complexes or the ligand for 3 h in the same medium. Afterward, the media was changed to an XF DMEM Assay Medium (pH 7.4) supplemented with 2 mM glutamine and cells were incubated in a non-CO_2_ incubator at 37 °C for 1 h before performing the assay. The Seahorse XFe24 Analyzer was calibrated and the assay was performed using glycolytic stress test assay protocol as suggested by the manufacturer (Agilent Technologies, Santa Clara, CA, USA). Sequential injection of glucose (10 mM final), oligomycin (1 μM final), and 2-deoxy-d-glucose (50 mM final) was performed, and three measurements were executed after each injection with 2 min of mixing in between measurements.

#### Statistical Analysis

All the experimental data reported are the mean, with their respective SD, of at least three experiments. Comparisons between two groups were performed using non-paired two-tailed Student’s *t* test, and analysis of variance was performed with Tukey–Kramer method utilizing INSTAT 3.3 (Graph-Pad) software. A *p* value <0.05 was considered significant.

### Computational Methods

#### Docking

First, we optimized the geometry of CuTAML and AuTAML compounds. Based on solution stability data available in the literature, the Cu(II) ion in CuTAML was coordinated to two hydroxyl ions, in addition to the TAML ligand, while the coordination sphere of Au(III) was completed by a chloride ion and a hydroxyl ion.^63^ Geometry optimization was performed in Gaussian^100^ with the B3LYP functional.^101,102^ Basis set 6-31G*^103^ was employed for CuTAML and LanL2DZ^104^ for AuTAML. The compounds were then rigidly docked into estrogen receptor α ligand binding domain (ERα, PDB ID: 3ERT) in the antagonist conformation.^105^ The crystal structure of ERα contains 4-hydroxytamoxifen, which was removed to generate the apo form. Docking was performed with Glide using the extra precision (XP) protocol.^106^ Docking for TAML was performed accordingly. However, to take into account ionization and stereoisomerization of the compounds, the Ligprep tool from the Schrodinger Suite (Schrödinger, LLC, New York, NY, 2021) was used for ligand preparation. After docking, best scoring poses were subjected to molecular dynamics simulations. For molecular dynamics simulations of ERα in complex with 4-hydroxytamoxifen, the crystal structure of the complex was used as the starting point (PDB ID: 3ERT) and no docking was performed.

#### System Setup

System setup was performed with the TLEAP tool from the AmberTools22 package.^107^ Studied systems were first placed in a simulation box. The box size was selected such that the distance between the protein and box edge was at least 12 Å. Next, the system was solvated with TIP3P water. Na^+^ and Cl^–^ were added in the simulation box to achieve electroneutrality. Simulations were performed using the Amber force field ff14SB.^108^ The Na^+^ and Cl^–^ ions were described using Li and Merz 12–6 ion parameters.^109^ In the case of thioredoxin reductases (TrxR, PDB ID: 2J3N),^110^ AuTAML was covalently linked to selenocysteine (SeCys498). To obtain force field parameters for CuTAML, AuTAML, and AuTAML-SeCys, force constants and Merz–Kollman-restrained electrostatic potential (RESP) charges were calculated with Gaussian09^100^ using the Metal Center Parameter Builder (MCPB) workflow.^111^

#### Classical MD

Classical MD simulations were performed with the GROMACS software package (version 2020.3).^112^ After minimization, simulated systems were equilibrated in a canonical (NVT) ensemble (*T* = 300 K) for 5 ns using periodic boundary conditions and position restraints (1000 kJ/mol nm^2^) on the protein and the docked compound. Next, equilibration proceeded for another 5 ns in the isothermal–isobaric (NPT) ensemble in order to equilibrate the system at 1 bar, while maintaining position restraints. The restrains were slowly released, and trajectories were recorded for another 300 ns (2 fs time step). Newton’s equations of motion were integrated with the leap-frog algorithm. Electrostatic interactions were evaluated using the Particle Mesh Ewald method.^113^ Temperature and pressure were controlled with v-rescale thermostat^114^ (τ_*T*_ = 0.1 ps) and Parrinello–Rahman barostat^115^ (τ_*p*_ = 2 ps), respectively.

#### Binding Free Energy Calculations

Binding free energies were calculated with the molecular mechanics with the Poisson–Boltzmann and surface area solvation (MM-PBSA) method.^116^ Calculations were performed with the MMPBSA.py program from the AmberTools22 package.^117^ Binding free enthalpies were calculated over 100 frames, extracted at regular intervals from the MD trajectories. The non-polar solvation free energy was composed of the cavity term and the dispersion term (inp = 2). Internal and external dielectric constants were set to 4.0 and 80.0, respectively. Salt concentration was 0.15 M. The conformational entropic contribution of the free energy was not taken into account, as it was suggested that this term does not improve the quality of the results using the MM-PBSA.^118,119^

## Supporting Information Available

Scheme S1: synthetic pathway to TAML as reported by Hey-Hawkins and coworkers; Figure S1: comparison of AuTAML and TAML ^1^H NMRs collected in CD_3_CN; Figure S2: ^1^H NMR spectrum of AuTAML collected in CD_3_CN; Figure S3: ^13^C NMR spectrum of AuTAML collected in CD_3_CN; Figure S4: HSQC NMR spectrum of AuTAML collected in CD_3_CN; Figure S5: COSY NMR spectrum of AuTAML collected in CD_3_CN; Figure S6: ESI-HR-MS spectrum of AuTAML; Figure S7: ESI-HR-MS spectrum of CuTAML; Figure S8: molecular structure of a AuTAML cation with a AuCl_2_ anion; Figure S9: UV–vis spectra of CuTAML in 1× PBS (pH 7.4) recorded over 24 h; Figure S10: UV–vis spectra of AuTAML (60 μM) in 1× PBS (pH 7.4) and UV–vis spectra recorded at *t* = 0 (blue trace) and 24 h (red trace); Figure S11: UV–vis spectra of AuTAML, CuTAML, and TAML in 1× PBS (pH 7.4); Figure S12: UV–vis spectra of AuTAML (60 μM) in 1× PBS (pH 7.4) with 2 equiv of GSH at 37 °C recorded at *t* = 0 (blue trace) and 24 h (red trace); Figure S13: ^1^H NMR spectra of AuTAML in CD_3_CN/D_2_O (80/20) at *t* = 0 (bottom trace) and 24 h (top trace); Figure S14: scheme of Seahorse analysis step by step; Figure S15: assessment of glycolysis in cells treated with the complexes or their ligand; Table S1: table of crystal data, data collection, and structure refinement of AuTAML (PDF)
Molecular formula strings (CSV)

## ABBREVIATIONS

BIAM: biotinylated iodoacetamide
Cyt c: cytochrome c
DHR: dihydrorhodamine 123
ΔΨ_m_: mitochondrial membrane potential
ECAR: extracellular acidification rate
ER: estrogen receptor
FCCP: carbonyl cyanide 4-(trifluoromethoxy) phenylhydrazone
GR: glutathione reductase
MeCN: acetonitrile
MD: molecular dynamics
MLM: mouse liver mitochondria
MTT: 3-[4,5-dimethylthiazol-2-yl]- 2,5-diphenyltetrazolium bromide
OCR: oxygen consumption rate
ROS: reactive oxygen species
PMSF: phenylmethylsulfonyl fluoride
RS: reserve capacity
SDS-PAGE: sodium dodecyl sulfate-polyacrylamide gel electrophoresis
SERM: selective estrogen receptor modulators
SERD: selective estrogen receptor degraders
TAML: 4-[1,1-bis(4-methoxyphenyl)but-1-en-2-yl]-2,2′-bipyridine
TMRM: tetramethyl rhodamine methyl ester
TrxR: thioredoxin reductase
XRD: X-ray diffraction
