# Supplementary material for: Exploring the Anticancer Activity of Tamoxifen-Based Metal Complexes Targeting Mitochondria
Source: J Med Chem. 2023 Jul 6;66(14):9823–41. doi: 10.1021/acs.jmedchem.3c00617 (PMC10388301; doi:10.1021/acs.jmedchem.3c00617)
Supplement: Supplementary file 1 — jm3c00617_si_001.pdf [file jm3c00617_si_001.pdf]

Supporting Information

Exploring the Anticancer Activity of Tamoxifen-based Metal Complexes Targeting Mitochondria

Valeria Scalcon,[a] Riccardo Bonsignore,[b] Jana Aupič,[c] Sophie R. Thomas,[d] Alessandra Folda,[a] Alexandra A. Heidecker,[e] Alexander Pöthig,[e] Alessandra Magistrato,[c,] Angela Casini[d,*] and Maria Pia Rigobello [a,*]*

[a] Department of Biomedical Sciences, University of Padova, Via Ugo Bassi 58/b, 35131 Padova, Italy.

[b] Dipartimento di Scienze e Tecnologie Biologiche, Chimiche e Farmaceutiche, Università degli Studi di Palermo, Viale delle Scienze, Edificio 17, 90128 Palermo, Italy.

[c] National Research Council of Italy Institute of Materials (CNR-IOM) C/o SISSA, Via Bonomea 265, 34136, Trieste, Italy.

[d] Chair of Medicinal and Bioinorganic Chemistry, Department of Chemistry, School of Natural Sciences, Technical University of Munich, Lichtenbergstraße 4, D-85748 Garching b. München, Germany.

[e] Catalysis Research Center & Department of Chemistry, Chair of Inorganic and Metal-Organic Chemistry, School of Natural Sciences, Technical University of Munich, Ernst-Otto-Fischer Str. 1, D-85748 Garching b. München, Germany.

[*]corresponding authors:

Alessandra Magistrato, Email: alessandra.magistrato@sissa.it

Supporting Information

Angela Casini, Email: angela.casini@tum.de

Maria Pia Rigobello, Email: mariapia.rigobello@unipd.it

Supporting Information

SI content:

Scheme S1. Synthetic pathway to TAML as reported by Hey-Hawkins and coworkers.

Figure S1. Comparison of AuTAML and TAML ^{1}H NMRs collected in CD_3CN.

Figure S2. ^{1}H NMR spectrum of AuTAML collected in CD_3CN.

Figure S3. ^{13}C NMR spectrum of AuTAML collected in CD_3CN.

Figure S4. HSQC NMR spectrum of AuTAML collected in CD_3CN.

Figure S5. COSY NMR spectrum of AuTAML collected in CD_3CN.

Figure S6. ESI-HR-MS spectrum of AuTAML.

Figure S7. ESI-HR-MS spectrum of CuTAML.

Figure S8. Molecular structure of AuTAML cation with a $AuCl_2$ anion.

Figure S9. UV-vis spectra of CuTAML in 1x PBS (pH 7.4) recorded over 24 h.

Figure S10. UV-vis spectra of AuTAML (60 μM) in 1x PBS (pH 7.4) and UV-vis spectra recorded at t= 0 (blue trace) and 24 h (red trace).

Figure S11. UV-vis spectra of AuTAML, CuTAML and TAML in 1x PBS (pH 7.4).

Figure S12. UV-vis spectra of AuTAML (60 μM) in 1x PBS (pH 7.4) with 2 eq. of GSH at 37 °C recorded at t= 0 (blue trace) and 24 h (red trace).

Figure S13. ^{1}H NMR spectra of AuTAML in CD_3CN/D_2O (80/20) at t = 0 (bottom trace) and 24 h (top trace).

Figure S14. Scheme of Seahorse analysis step by step.

Figure S15. Assessment of glycolysis in cells treated with the complexes or their ligand.

Table S1. Table of crystal data, data collection and structure refinement of AuTAML.

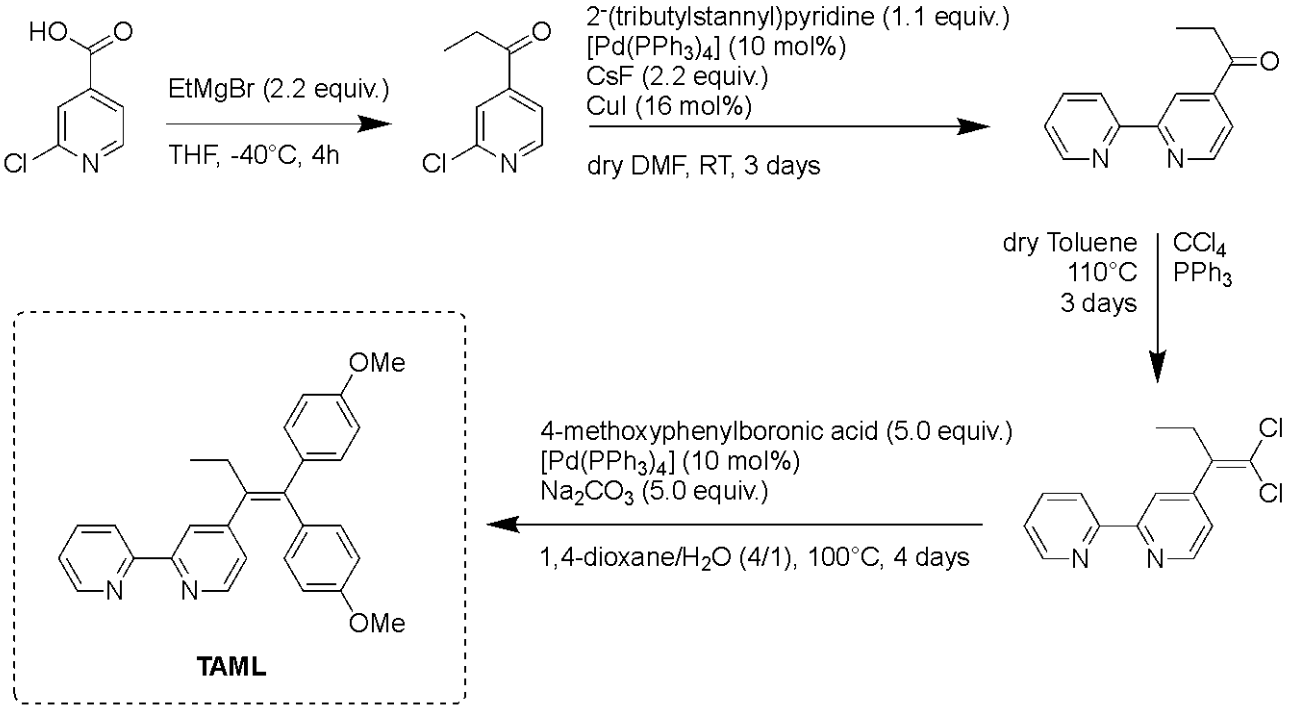

Scheme S1. Synthetic pathway to TAML as reported by Hey-Hawkins and coworkers. [37]

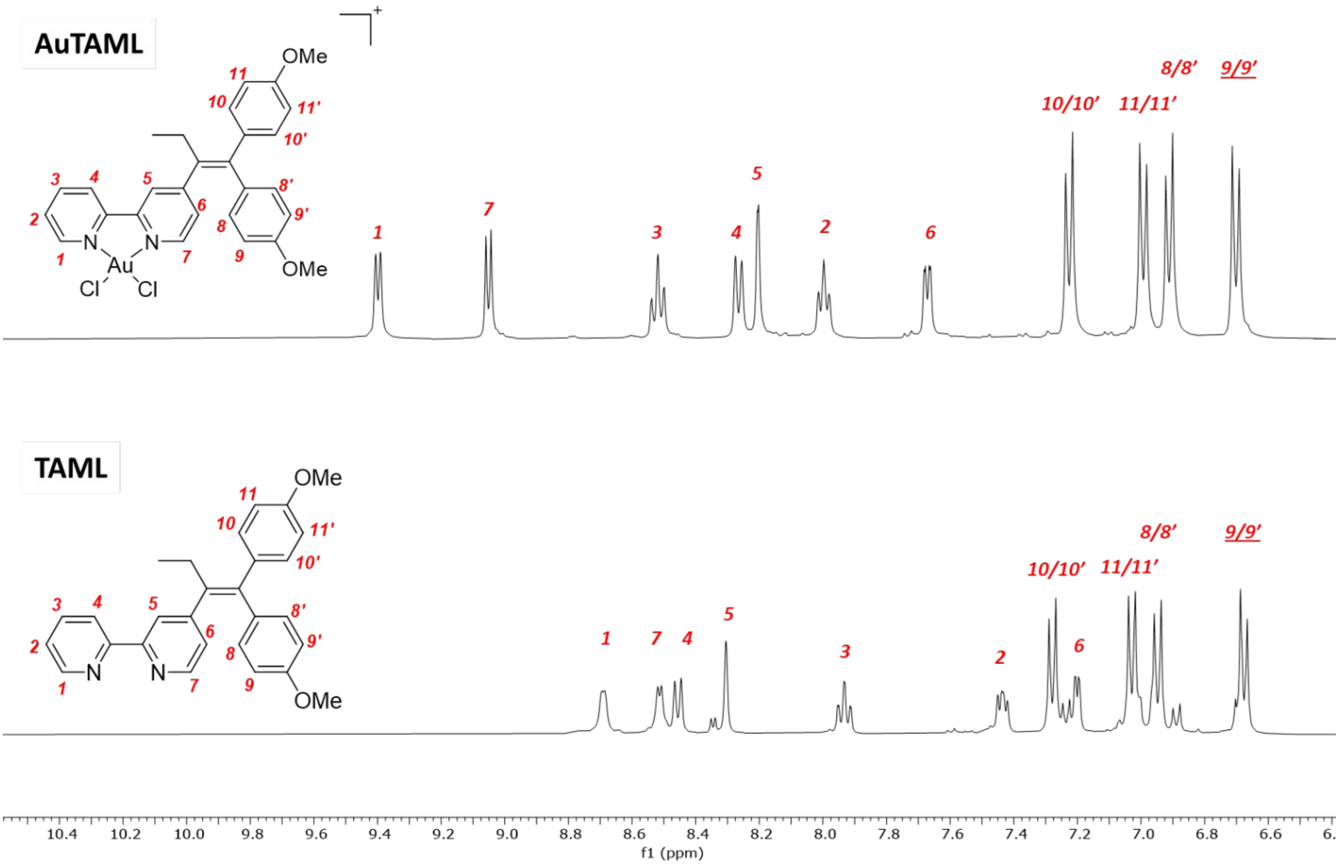

Figure S1. Comparison of AuTAML (above) and TAML (below) ^{1}H NMRs with respective proton assignments collected in CD_3CN.

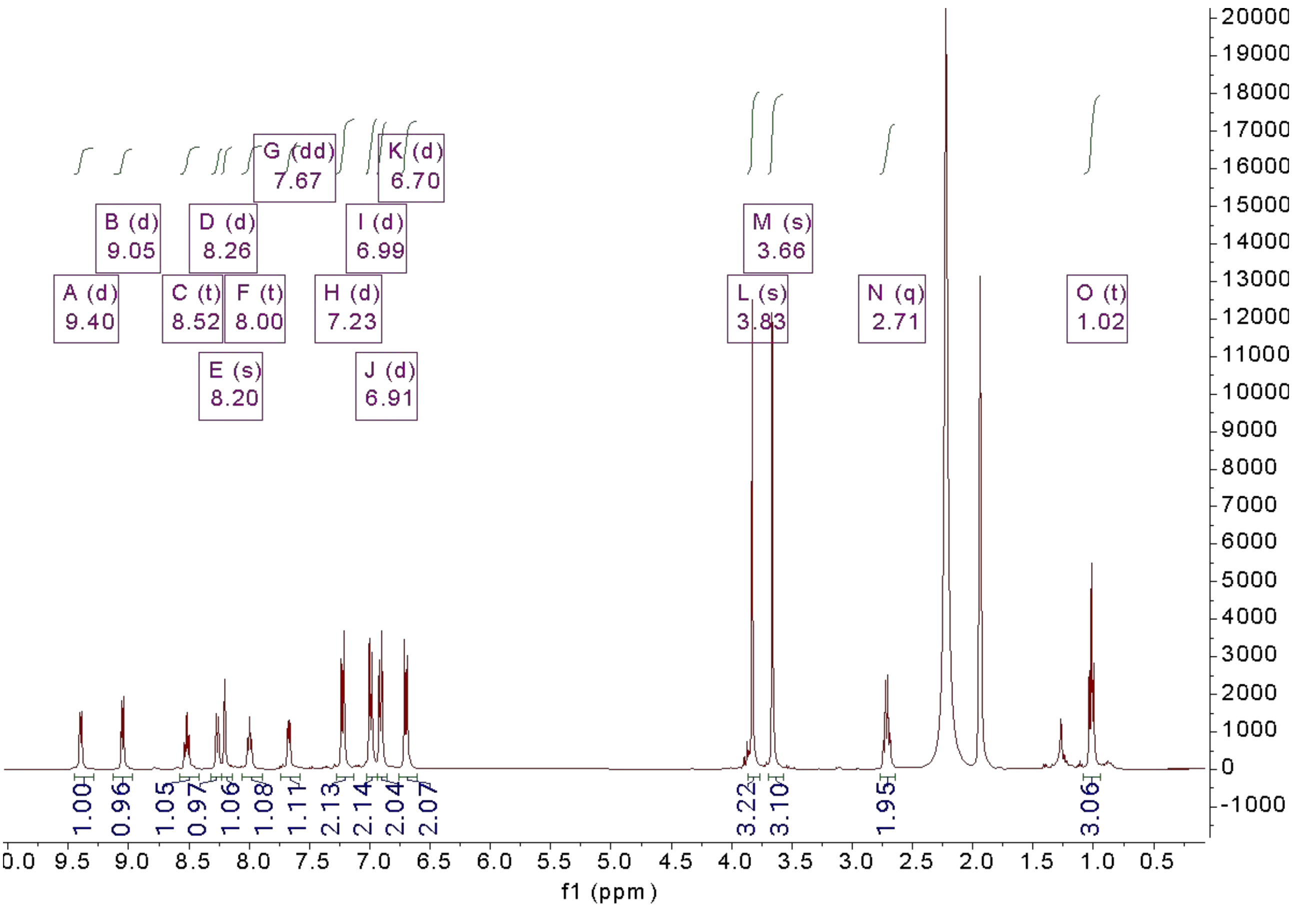

Figure S2. ^{1}H NMR spectrum of AuTAML collected in CD_3CN.

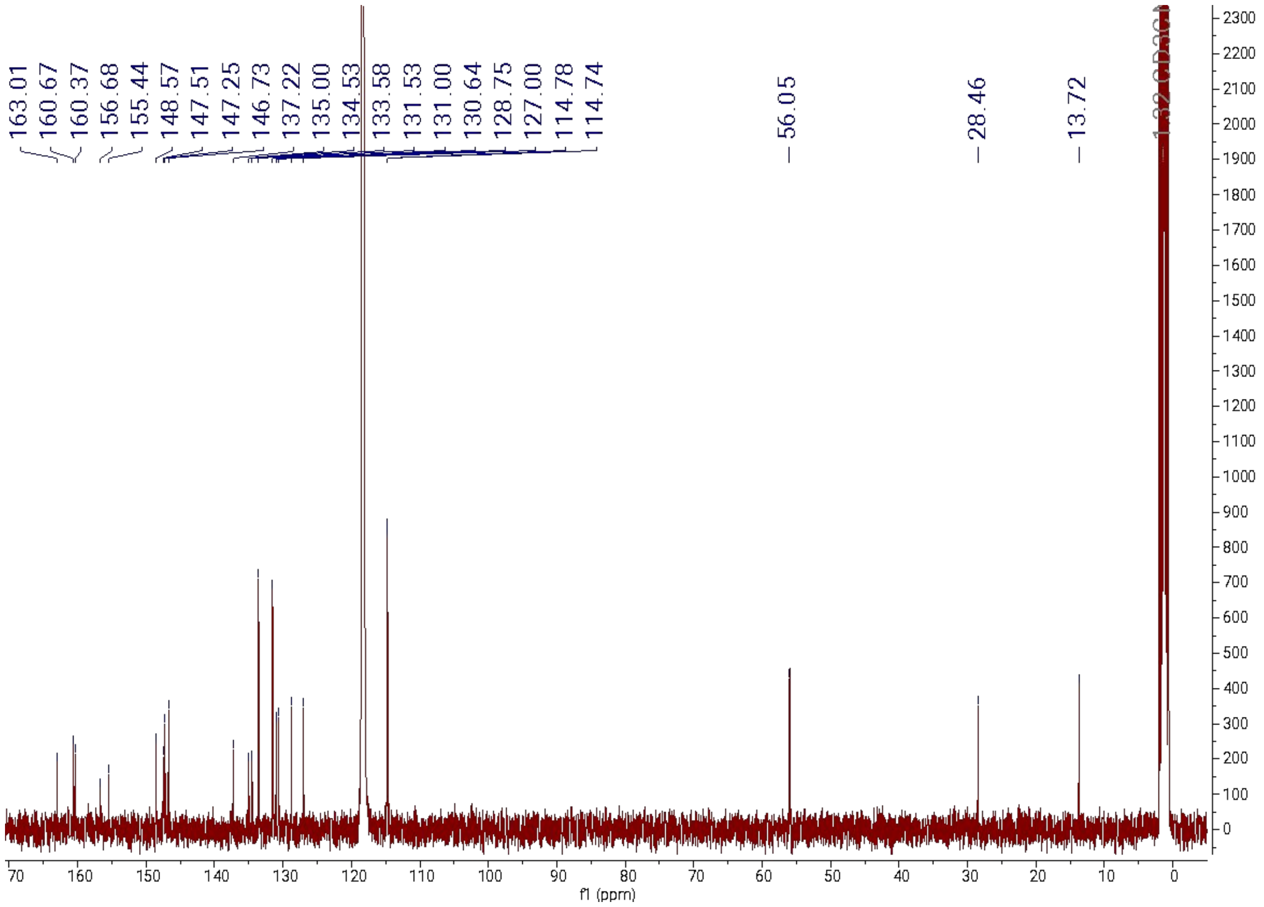

Figure S3. ^{13}C NMR spectrum of AuTAML collected in CD_3CN.

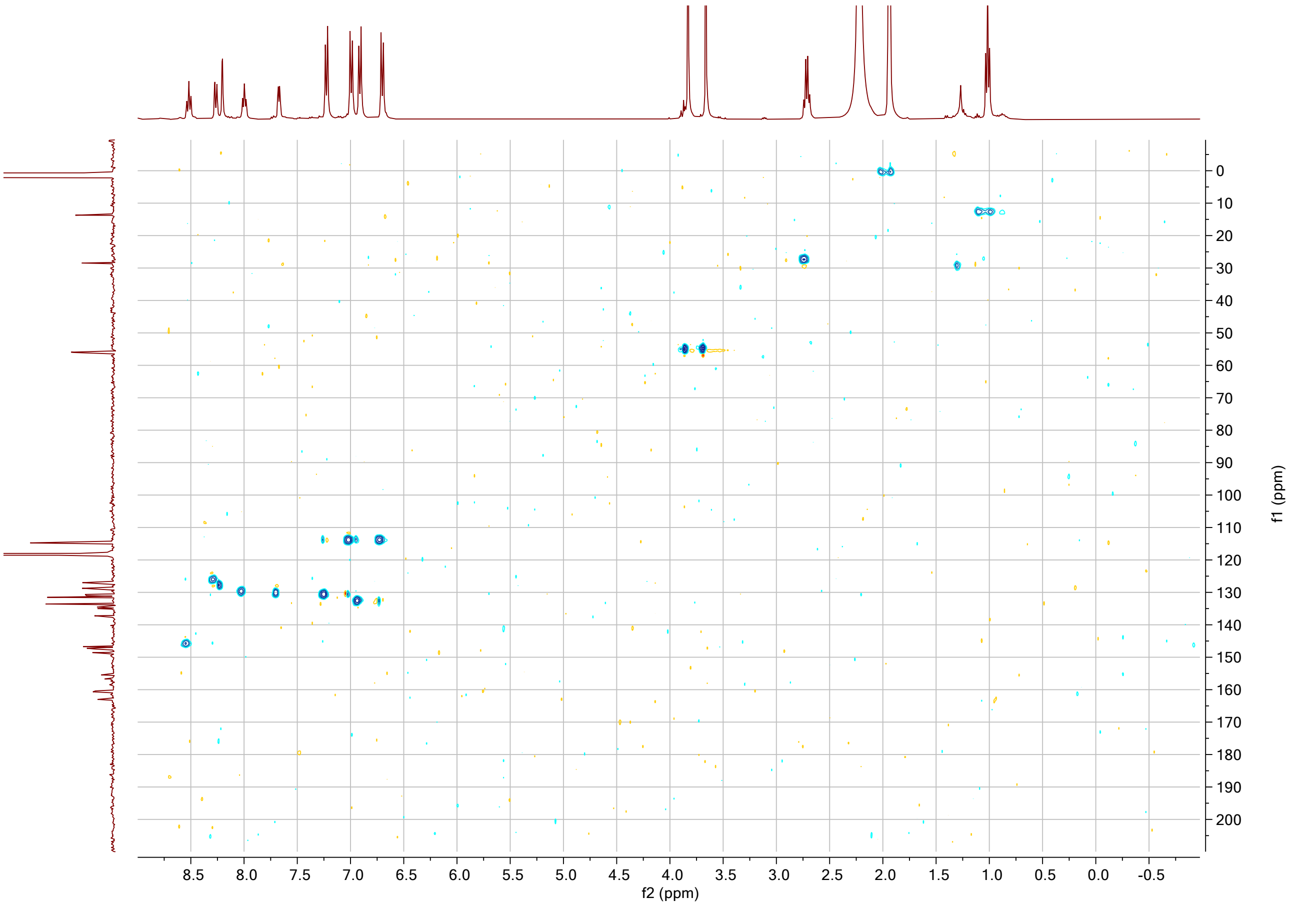

Figure S4. HSQC NMR spectrum of AuTAML collected in CD_3CN.

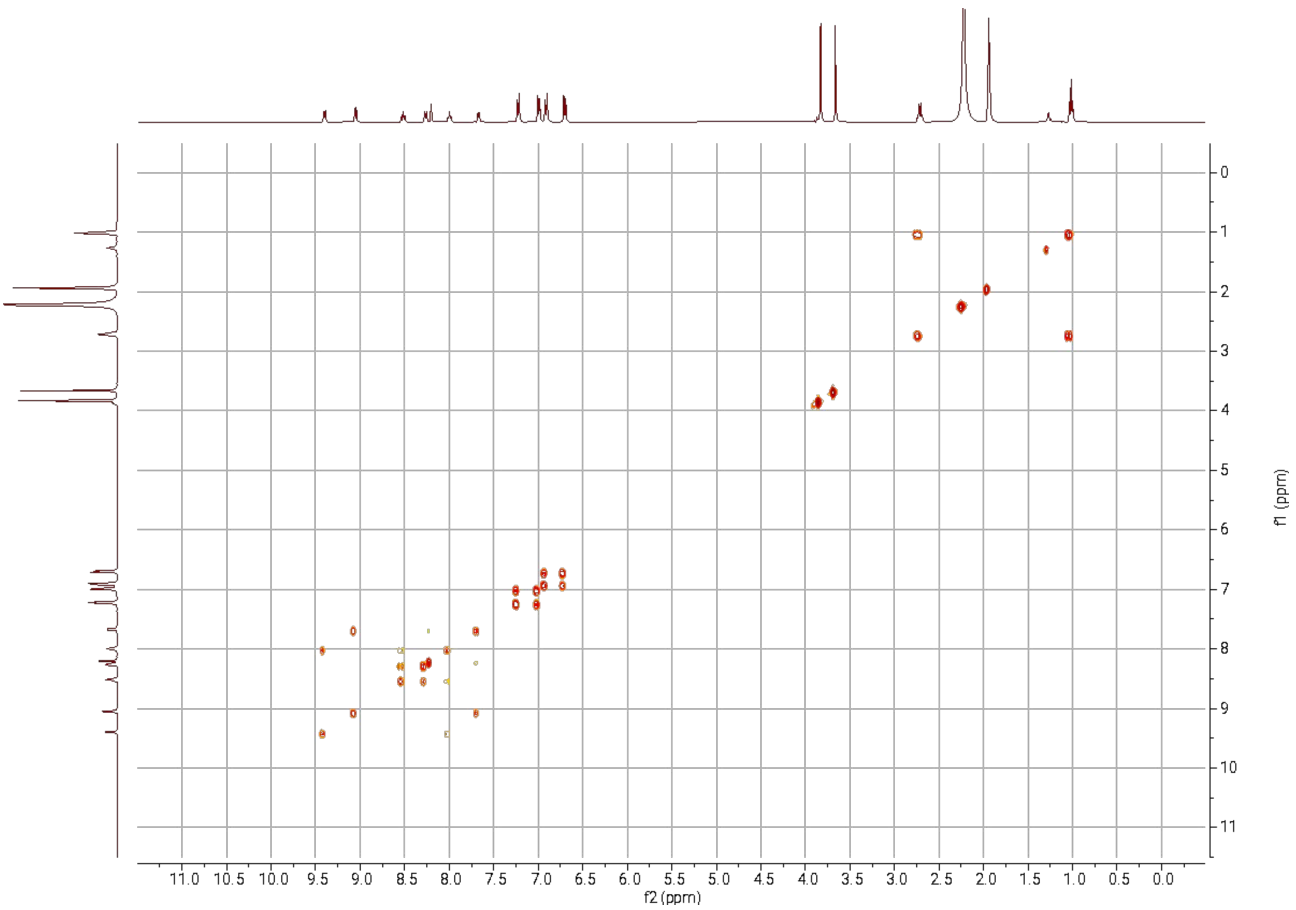

Figure S5. COSY NMR spectrum of AuTAML collected in CD_3CN.

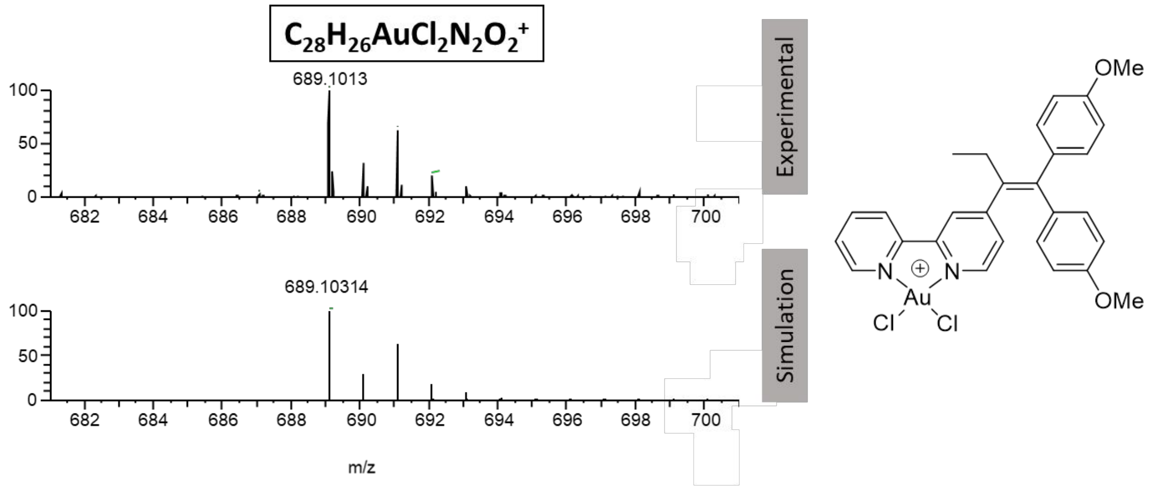

Figure S6. ESI-HR-MS spectrum of AuTAML.

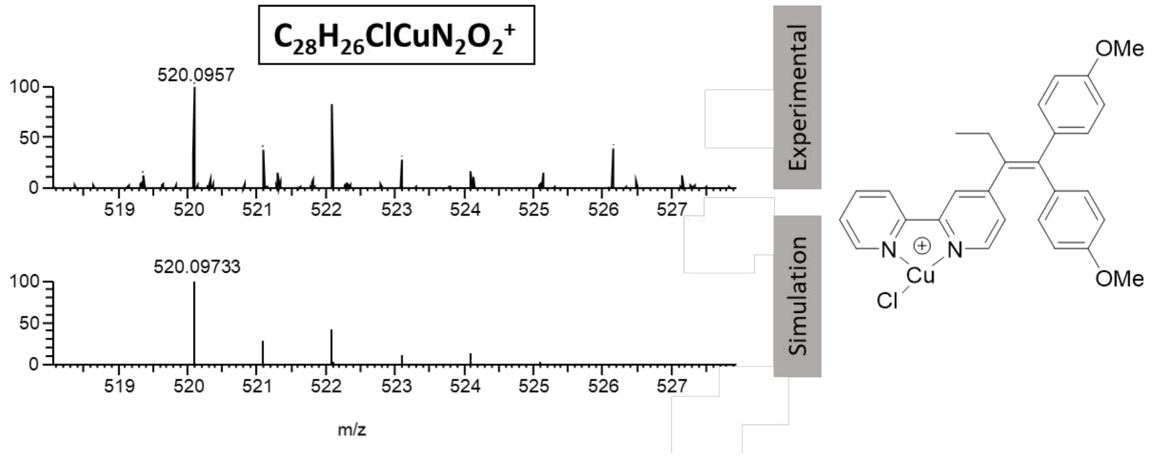

Figure S7. ESI-HR-MS spectrum of CuTAML.

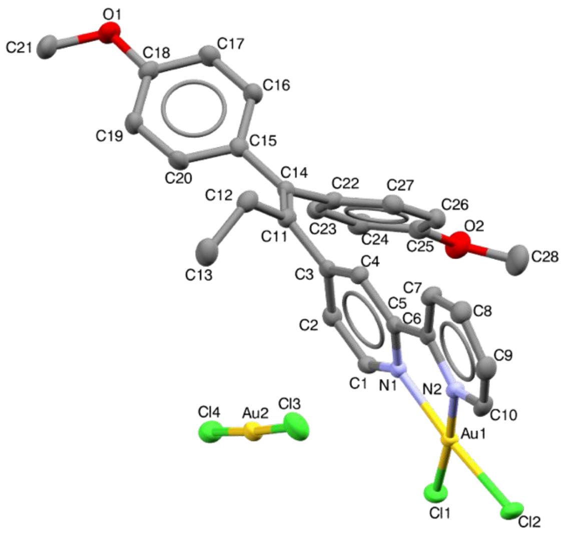

Figure S8. Molecular structure of AuTAML cation with a $AuCl_2^-$ anion with ellipsoids set at 50% probability level. Hydrogens have been omitted for clarity.

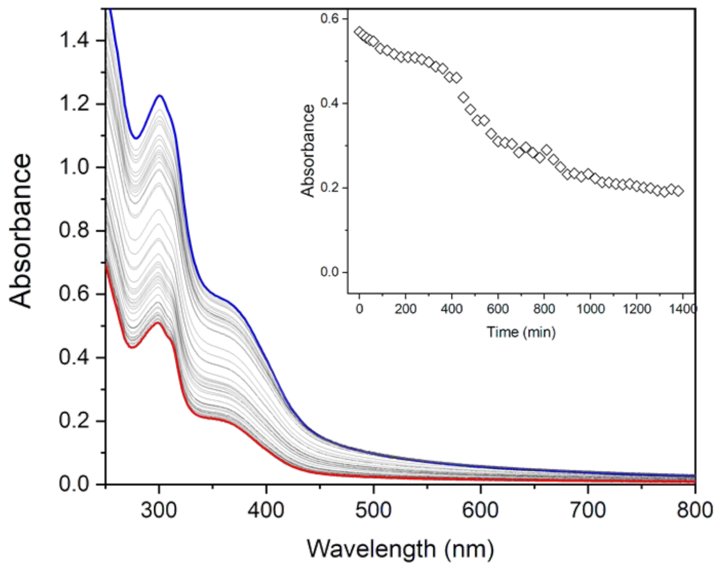

Figure S9. UV-vis spectra of CuTAML (60 μM) in 1x PBS (pH 7.4) recorded over 24 h. In the inset, evolution of the absorbance of CuTAML at 357 nm over time.

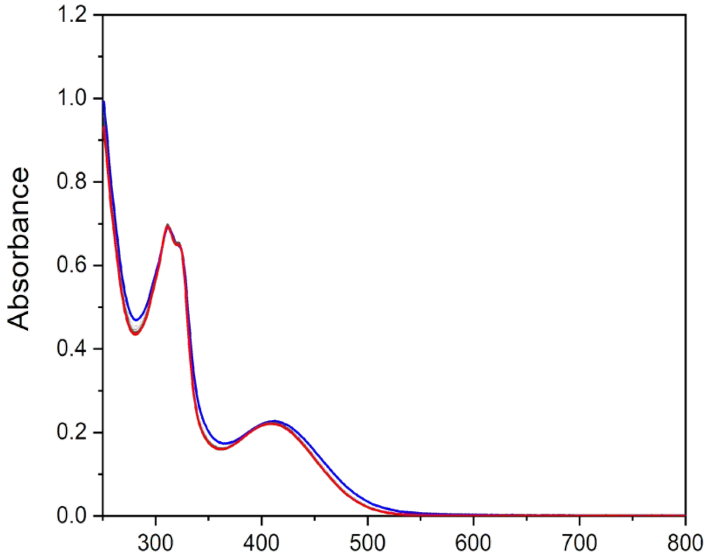

Figure S10. Stability studies by UV-vis absorption spectroscopy. UV-vis spectra of AuTAML (60 µM) in 1x PBS (pH 7.4) and UV-vis spectra recorded at t= 0 (blue trace) and 24 h (red trace).

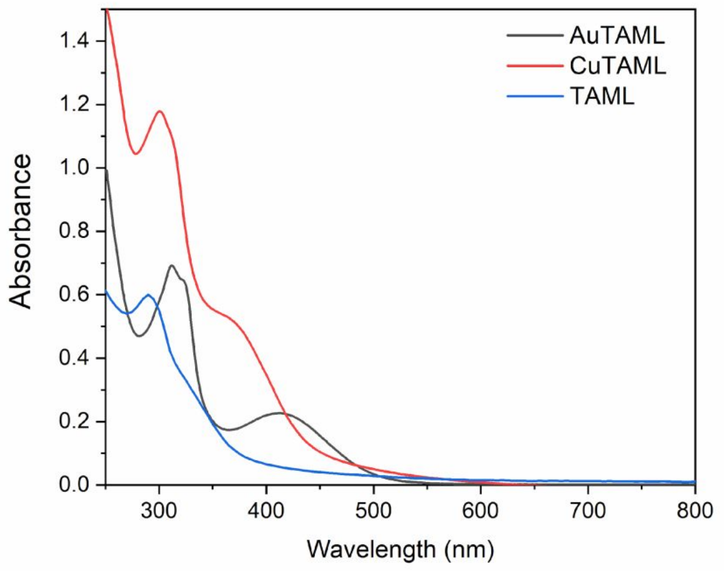

Figure S11. UV-vis spectra of AuTAML (60 μM, black), CuTAML (60 μM, red) and TAML (30 μM, blue) in 1x PBS (pH 7.4).

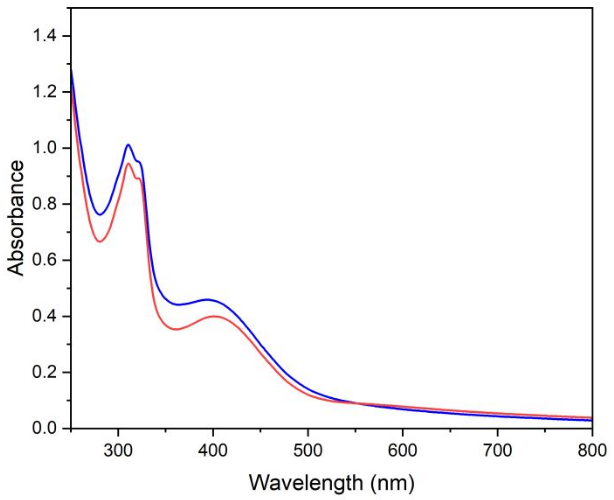

Figure S12. UV-vis spectra of AuTAML (60 μM) in 1x PBS (pH 7.4) with 2 eq. of GSH at 37 °C recorded at t= 0 (blue trace) and 24 h (red trace).

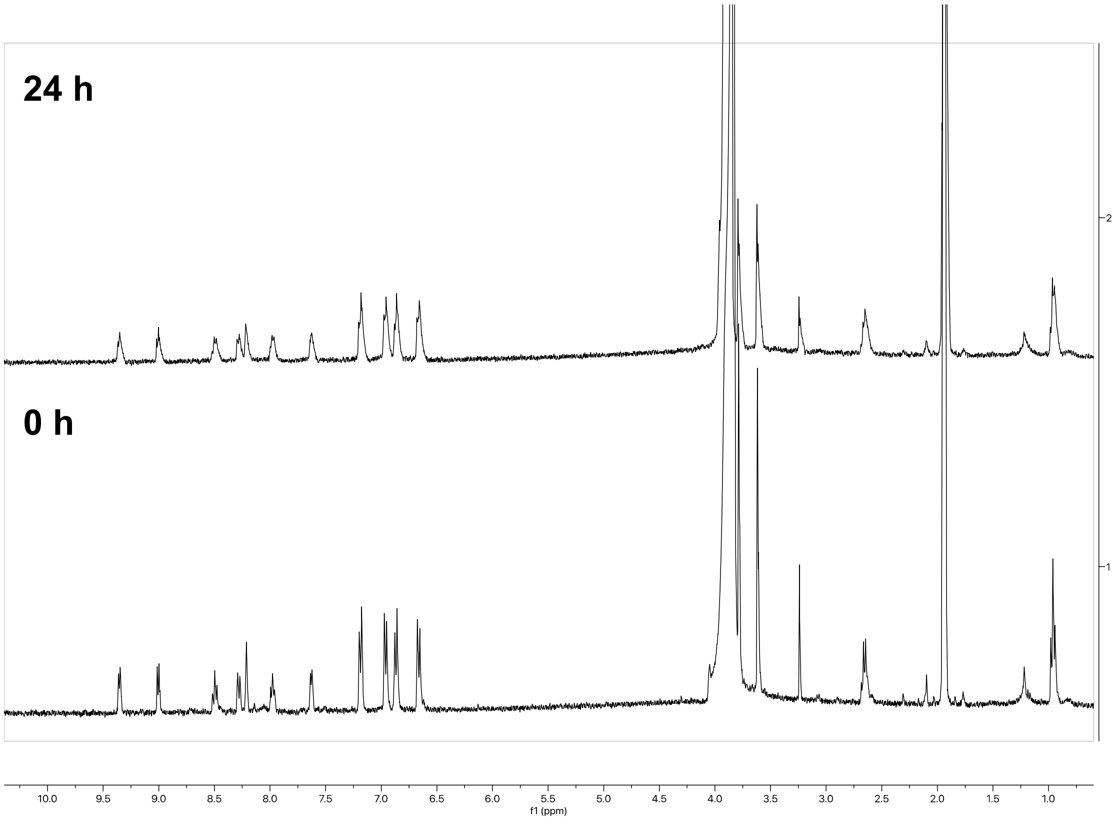

Figure S13. ^{1}H NMR spectra of AuTAML in CD_3CN/D_2O (80/20) at t = 0 (bottom trace) and 24 h (top trace).

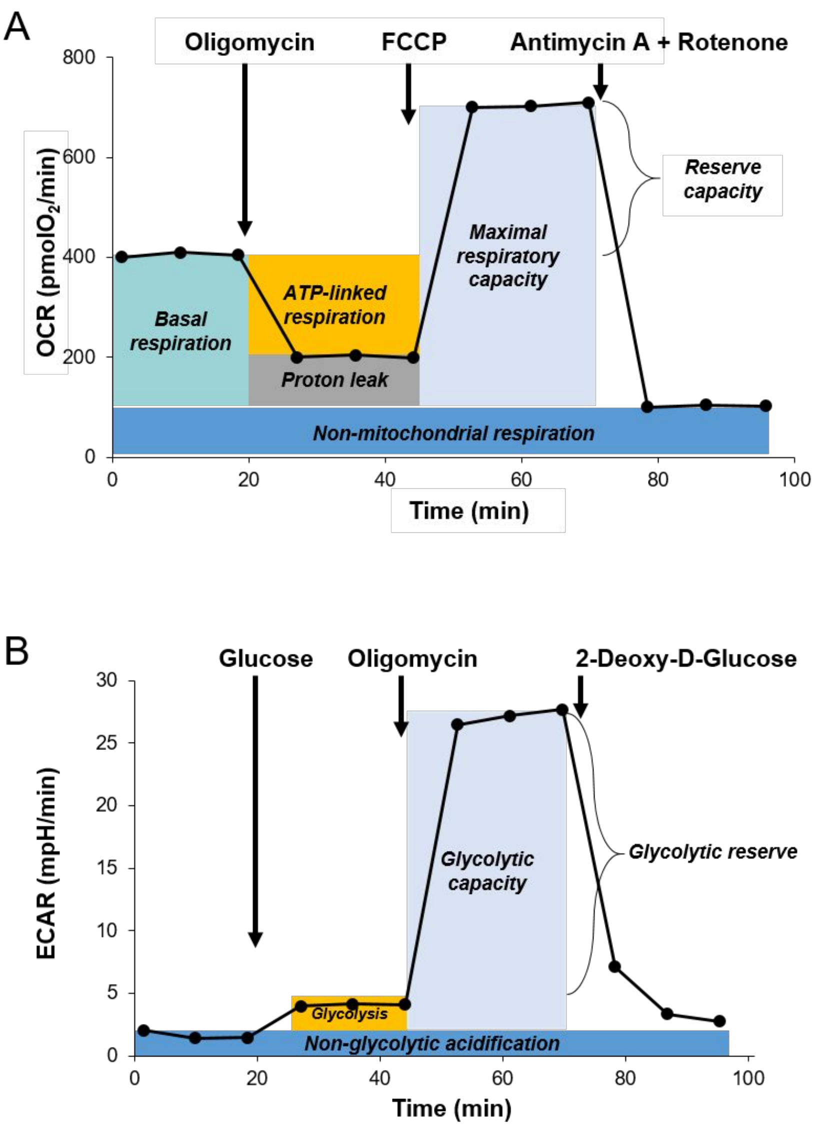

Figure S14. Scheme of Seahorse analysis step by step. A) OCR; **B)** ECAR.

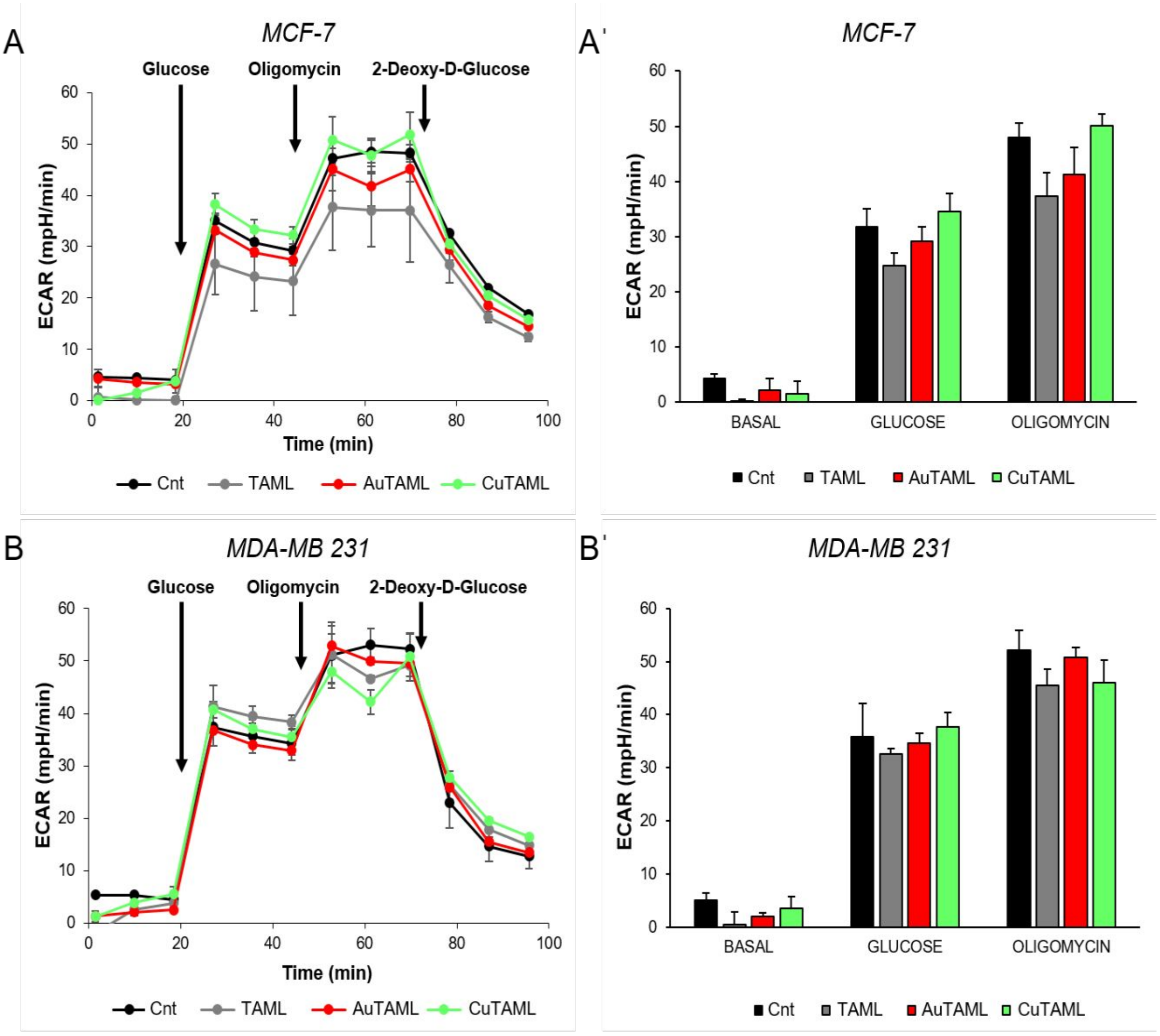

Figure S15. Assessment of glycolysis in cells treated with the complexes or their ligand.

Glycolysis was determined in both MCF-7 (**A, A'**) and MDA-MB 231 (**B, B'**) cells after treatment with 5 μM of the compounds for 3 h using the Seahorse Xfe24 analyser as described in the Experimental section. 2-DG: 2-deoxyglucose, glycolysis inhibitor. Mean values ± SD of 3 experiments are reported.

Supporting Information

Table S1. Table of crystal data, data collection and structure refinement of AuTAML.

Identification code	BonRi26_1
Chemical formula	$C_{28}H_{26}Au_2Cl_4F_0N_2O_2$
Formula weight	958.24
Temperature	134(2) K
Wavelength	0.71073 Å
Crystal size	0.040 x 0.061 x 0.167 mm
Crystal habit	clear dark orange needle
Crystal system	monoclinic
Space group	C 1 c 1
Unit cell dimensions	a = 8.9434(5) Å α = 90°
	b = 52.906(3) Å β = 122.338(2)°
	c = 7.0672(4) Å γ = 90°
Volume	2825.3(3) $Å^3$
Z	4
Density (calculated)	2.253 g/cm^3
Absorption coefficient	10.782 mm^{-1}
F(000)	1800
Diffractometer	Bruker D8 Venture
Radiation source	TXS rotating anode, Mo
Theta range for data collection	2.31 to 26.02°

Index ranges	-11<=h<=11, -64<=k<=64, -8<=l<=8
Reflections collected	57535
Independent reflections	5492 [R(int) = 0.0395]
Coverage of independent reflections	99.9%
Absorption correction	Multi-Scan
Max. and min. transmission	0.6720 and 0.2660
Structure solution technique	direct methods
Structure solution program	SHELXT 2018/2 (Sheldrick, 2018)
Refinement method	Full-matrix least-squares on F^2
Refinement program	SHELXL-2018/3 (Sheldrick, 2018)
Function minimized	Σ w(F_o^2 - F_c^2)^2
Data / restraints / parameters	5492 / 2 / 347
Goodness-of-fit on F^2	1.074
Δ/σ_{max}	0.011
	5448
Final R indices	data; R1 = 0.0136, wR2 = 0.0296
	I>2σ(I)

	all data R1 = 0.0138, wR2 = 0.0297
**Weighting scheme**	$w=1/[\sigma^2(F_o^2)+(0.0119P)^2+0.6545P]$ where $P=(F_o^2+2F_c^2)/3$
**Absolute structure parameter**	0.0(0)
**Largest diff. peak and hole**	0.529 and -1.006 eÅ^{-3}
**R.M.S. deviation from mean**	0.109 eÅ^{-3}
